# Mapping the soil microbiome functions shaping wetland methane emissions

**DOI:** 10.1128/msystems.00680-25

**Published:** 2026-06-01

**Authors:** Mikayla A. Borton, Angela M. Oliverio, Adrienne B. Narrowe, Jorge A. Villa, Christian Rinke, David W. Hoyt, Pengfei Liu, Bridget B. McGivern, Emily K. Bechtold, Jared B. Ellenbogen, Rebecca A. Daly, Garrett J. Smith, Jordan C. Angle, Rory M. Flynn, Andrew P. Freiburger, Katherine B. Louie, Brooke Stemple, Trent R. Northen, Christopher Henry, Christopher S. Miller, Timothy H. Morin, Gil Bohrer, Kelly C. Wrighton

**Affiliations:** 1 Department of Food Science and Human Nutrition, Colorado State University 3447, Fort Collins, Colorado, USA; 2 Department of Soil and Crop Sciences, Colorado State University 3447, Fort Collins, Colorado, USA; 3 Department of Biology, Syracuse University 2029, Syracuse, New York, USA; 4 School of Geosciences, University of Louisiana at Lafayette 4365, Lafayette, Louisiana, USA; 5 Department of Microbiology, University of Innsbruck 27255, Innsbruck, Austria; 6 Environmental Molecular Sciences Laboratory, Pacific Northwest National Laboratory 6865, Richland, Washington, USA; 7 Center for Pan-third Pole Environment, Lanzhou University 12426, Lanzhou, China; 8 Department of Microbiology, The Ohio State University 2647, Columbus, Ohio, USA; 9 Data Science and Learning, Argonne National Laboratory 1291, Lemont, Illinois, USA; 10 Lawrence Berkeley National Lab 1666, Berkeley, California, USA; 11 Lawrence Berkeley National Laboratory, DOE Joint Genome Institute 1666, Berkeley, California, USA; 12 Department of Integrative Biology, University of Colorado Denver 12226, Denver, Colorado, USA; 13 Department of Environmental Resources Engineering, State University of New York College of Environmental Sciences and Forestry, Syracuse, New York, USA; 14 Department of Civil, Environmental & Geodetic Engineering, The Ohio State University 2647, Columbus, Ohio, USA; Los Alamos National Laboratory 5112, Los Alamos, New Mexico, USA

**Keywords:** genome, MAG, metagenomics, metatranscriptomics, global change, greenhouse gases, methanogen

## Abstract

Accounting for only 8% of Earth’s land cover, freshwater wetlands remain the foremost contributors to global methane emissions. Yet the microorganisms and processes underlying methane emissions from wetland soils remain poorly understood. Over a five-year period, we surveyed the microbial membership and *in situ* methane measurements from over 700 samples in one of the most prolific methane-emitting wetlands in the United States. We constructed a catalog of 2,502 metagenome-assembled genomes (MAGs), with more than half of the 70 bacterial and archaeal phyla sampled containing novel lineages. Integration of these data with 133 soil metatranscriptomes provided a genome-resolved view of the biogeochemical specialization and versatility expressed over wetland soil spatial and temporal gradients. Centimeter-scale depth differences best explained patterns of microbial community structure and transcribed functionalities, even more than land cover or temporal information. Moreover, while extended flooding restructured soil redox, this perturbation failed to reconfigure the transcriptional profiles of methane-cycling microorganisms, contrasting with theoretically expected responses to hydrological perturbations. Co-expression analyses, coupled with depth-resolved methane measurements, revealed the metabolisms and trophic structures most predictive of methane hotspots. Mapping the spatiotemporal transcriptional patterns on this compendium of biogeochemically classified soil-derived genomes begins to untangle the microbial carbon, energy, and nutrient processing contributing to wetland methane production.

**IMPORTANCE:**

Soil microbial ecology is increasingly recognized as essential to climate mitigation, but realizing its full potential requires shifting from static genome inventories to dynamic assessments of microbial activity. This study shows that methane-cycling microbes exhibit stable, depth-stratified expression patterns, even in response to major redox and flooding shifts, undermining assumptions that water-table manipulations common in wetland management can alone reduce methanogenesis. Instead, methane cycling is shaped by spatially organized, transcriptionally active networks involving not only methanogens but also methanotrophs, fermenters, and iron reducers. These findings expose the limitations of genome-only models and highlight the need for soil diagnostics that capture *in situ* activity. Together, we provide a foundation for developing activity-based microbiome tools, embedding microbial functions into Earth system models, and designing interventions that move beyond “single-lever” strategies and instead work with the structure and dynamics of microbial communities as complex, layered systems.

## INTRODUCTION

Wetlands are characterized as waterlogged soils, rich in organic matter, with low concentrations of oxygen in the porewater. Consequently, the anoxic microbial decomposition of this soil organic matter leads to the production of biogenic methane (CH_4_). As such, wetland soils are the largest and most variable source of this potent greenhouse gas, accounting for nearly a third of annual CH_4_ emissions (1–4). Despite substantial advances in identifying wetland microbial communities, metabolic pathways, and environmental controls regulating the conversion of soil organic carbon to CH_₄_ (5–12), uncertainties remain in linking genome-resolved microbial traits to process-level methane flux across spatial and temporal scales. This knowledge gap hinders the incorporation of microbial processes into land surface and biogeochemical models, impeding the accurate management and predictions of greenhouse gas fluxes from wetlands (3, 13, 14).

Over the last decade, metagenomic sequencing approaches have described the microbial community membership and function associated with CH_4_ production across a range of habitats. While other high-CH_4_-emitting systems, such as wastewater (5), ruminants (15), or subsurface habitats (16), have received considerable genomic scrutiny, wetland soils remain relatively underexplored in terms of microbial genomic sampling. Of the handful of genome-resolved studies from freshwater wetlands, nearly all are focused on organic soils from northern peatlands (10, 17–19). One notable study, from thawing permafrost in Sweden, yielded ~1,500 metagenome-assembled genomes (MAGs) (10), serving as the stand-alone microbial catalog for all wetland soils. This means that the highest CH_4_-contributing wetland types, temperate and tropical marshes, have only a few genome-resolved studies to date (6, 20, 21). These studies emphasized the roles of a limited number of microorganisms to specific biogeochemical processes (6, 20–22), such as methanotrophy, yet failed to inventory the overall composition, function, and interconnected metabolic pathways driving CH_4_ production across diverse wetland gradients.

To bridge this knowledge gap, we undertook a comprehensive sampling within a temperate freshwater marsh, aiming to elucidate the spatiotemporal variations in the microbiome. This freshwater wetland was chosen due to its exceptionally high CH_4_ fluxes, exceeding 10 times the median flux observed in wetlands with similar annual temperature ranges (23, 24), and because of its susceptibility to climate-induced flooding events influenced by fluctuating Lake Erie water levels (25). Leveraging more than 5 terabase pairs of sequencing, we reconstructed thousands of microbial genomes and integrated this data with soil metatranscriptomes, geochemistry, and greenhouse gas porewater measurements and fluxes.

Our primary objective was to resolve the microbial membership, expressed metabolisms, and metabolic interactions underlying methane production in a temperate freshwater wetland using a genome-resolved, multi-omics framework. Specifically, we aimed to expand genomic representation of methane-cycling lineages, linking their microbial activity and community interactions to methane concentrations across space and time. Here, we delineate the expressed membership and metabolisms of the microbial community, shedding light on the biogeochemical contributions of hundreds of understudied lineages. These findings offer new perspectives on the stability and interactions of metabolic guilds driving CH_4_ production in temperate freshwater wetland ecosystems.

## RESULTS

### Sampling microorganisms from wetland soils

**Fig 1:**
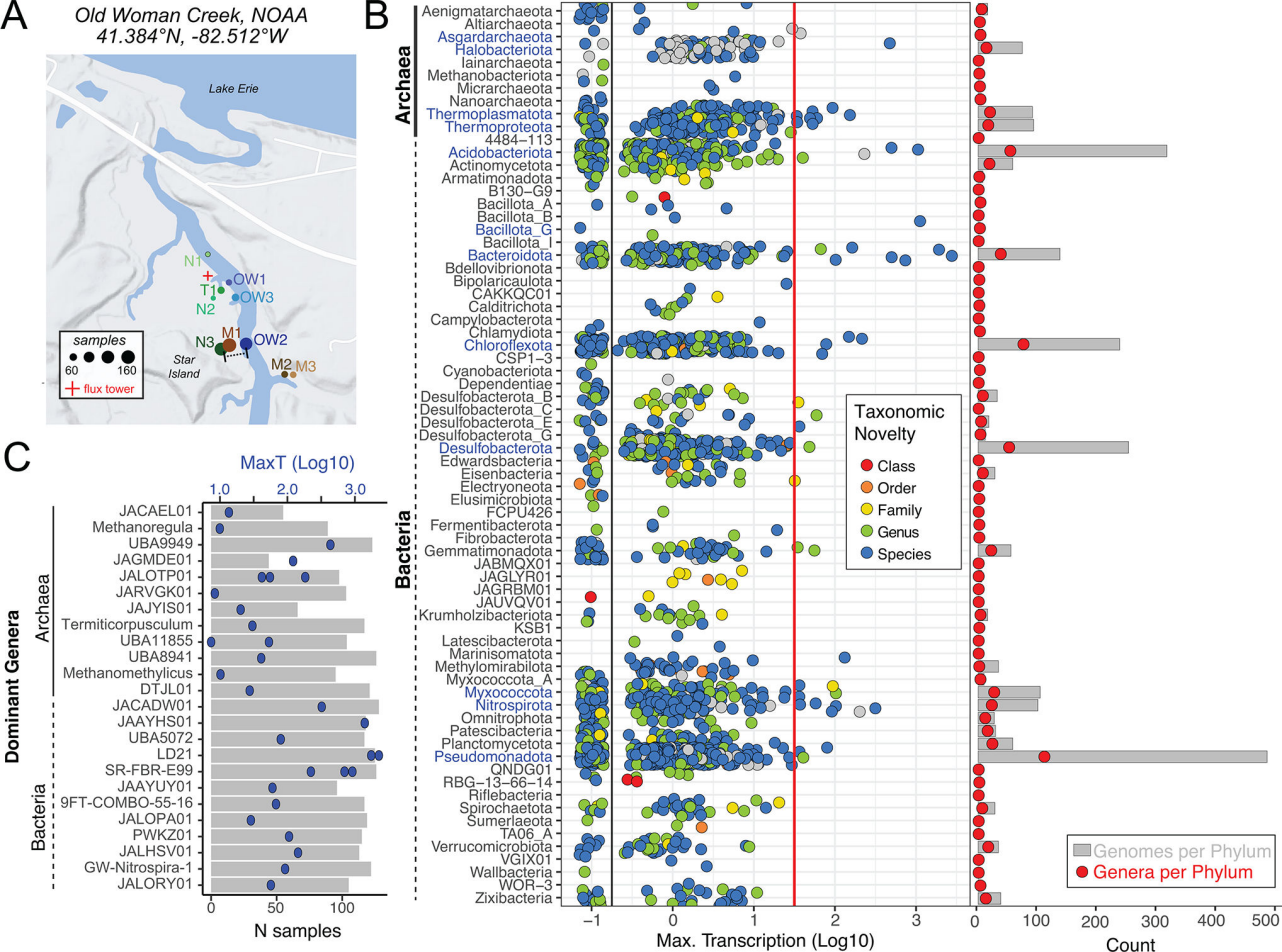
Freshwater wetland soils host thousands of taxonomically diverse MAGs that recruit metatranscripts. (**A**) Old Woman Creek (OWC) is located on the southern part of Lake Erie and is the highest U.S. methane-emitting wetland in the AmeriFlux Network. A site map of the OWC freshwater wetland shows where the 700 samples were collected from 10 major sampling sites that were historically defined as emergent vegetation, either *Nelumbo* (N1-3) or *Typha* (T1), mud cover (M1-3), or location in open water channel (OW1-3). Sites were sampled over multiple years and depths at 5 cm intervals from 0 to 30 cm, with sampling density proportional to circle size; 52 is the minimum sampling per location (Fig. S1). (**B**) The taxonomic distribution of the 2,502 MAGs is summarized at the phylum level. Blue text indicates the phylum contains a top 12 dominant genus (shown in panel **C**). Each point represents a MAG and is colored by taxonomic novelty. Points are distributed by their log10 maximum transcription across the 133 metatranscriptome samples. MAGs to the right of the black line were considered positive for sufficient transcript recruitment (*N* = 1,948). MAGs to the right of the red line had the greatest transcription (log10 max. transcription > 1.5). The gray bar chart to the right summarizes the number of genomes per phylum recovered, and red points overlaid represent the number of genera recovered within each phylum, using the same *x*-axis. (**C**) The most transcriptionally dominant archaeal and bacterial MAGs (top 15 per domain) are ranked using a composite metric that equally weighted maximum transcript abundance (MaxT) and occurrence across samples, grouped by genus. Gray bar indicates metatranscriptome prevalence (gray bar), or the number (N) of the 133 samples where metatranscripts were recruited to the MAG for that genus. The axis is shown at the bottom (N samples). The blue dot indicates the maximum transcription from panel **B**, with the axis (MaxT, Log10) shown at the top of the graph.

As a model wetland, we selected the highest methane-emitting wetland in the AmeriFlux network (site ID US-OWC), an investigator network with tailored instrumentation, data processing, and standardized procedures for flux measurements (24, 26–28). The Old Woman Creek (OWC) wetland, located in Ohio, USA, was instrumented to measure wetland CH_4_ at various spatiotemporal scales (Fig. S1), facilitating a detailed assessment of the microbial processes that impact soil CH_4_ dynamics. Based on land cover types, we parsed the wetland into 10 patches (Fig. 1A). These patches included emergent vegetation*-Typha* (*n* = 1), emergent vegetation*-Nelumbo* (*n* = 3), standing freshwater or open water (*n* = 3), and temporal mud flats (*n* = 3). From selected sites, once per month during sampling campaigns, dialysis peepers measured soil CO_2_ and CH_4_ concentrations at ~3 cm depth increments up to 30 cm. This content was paired to surface chamber flux measurements for CO_2_ and CH_4_ and site-wide fluxes using the eddy covariance and a meteorological station. Surrounding the peepers, soil cores were pulled and extruded at 5 cm depth increments down to 25 cm, resulting in 705 samples for soil geochemical, metabolite, and microbial analyses. In summary, across the five-year sampling campaign (2013–2018), each one of these 10 ecological patch sites was sampled at least 52 times for paired microbial, soil geochemistry, flux, and porewater GHGs (Table S1).

We screened 671 of these collected soil samples with 16S rRNA amplicon sequencing for methanogen membership (Table S2), identifying 42 samples for deep metagenomic sequencing (Table S1). We generated up to 300 Gbp of sequencing per sample, resulting in 3.1 Tbp total metagenomic sequencing. Collectively, this effort doubled the amount of metagenomic sequencing from wetland soils available in public repositories (Fig. S1). Given that one of our objectives was to increase the recovery of methanogen genomes from soils, multiple assembly and binning methods targeted the recovery of these lineages (Fig. S1). This approach resulted in over 17,333 draft genomes, which were dereplicated into a final collection of 3,217 genomic representatives, with 2,502 of these constituting high- and medium-quality metagenome-assembled genomes (MAGs) (Fig. S1 and Table S3). To map which microbial genomes recruited transcripts and which metabolisms were expressed across sites, depths, seasons, and years, we obtained 2.7 Tbp of metatranscriptome sequencing from 133 soil samples with paired geochemical data (Table S4). This comprehensive multi-omics sampling across wetland gradients, including multiple land cover types, depths, and times, provides the first insights into the microbial structural and functional diversity residing in temperate freshwater wetland soils.

### Uncovering thousands of transcribed genomes from wetland soils

We reconstructed MAGs from 60 bacterial (*N* = 2,204 MAGs) and 10 archaeal (*n* = 298 MAGs) phyla that included 85 methanogen-related MAG representatives. Many MAGs recovered here were designated as newly sampled lineages at the genus level or higher, including genome representatives with unnamed classes (*n* = 4 MAGs), orders (*n* = 20 MAGs), families (*n* = 15 MAGs), and genera (*n* = 122 MAGs) (Table S3). In addition, hundreds of genomes were from poorly or previously uncharacterized phyla often denoted only by an alphanumeric identifier or placeholder name, such as QNDG01, CSP1-3, and KSB1 (Fig. 1B). In this wetland, the most represented phyla are the archaeal Halobacteriota (*n* = 74 MAGs) and the bacterial Pseudomonadota (*n* = 484; formerly Proteobacteria), Acidobacteriota (*n* = 316 MAGs), Desulfobacterota (*n* = 252), and Chloroflexota (*n* = 237 MAGs). The reconstruction of these thousands of genomes, many metabolically uncharacterized, highlights the vast, unsampled microbial diversity still residing within soils.

Approximately 75% of the reconstructed genomes (*n* = 1,948) recruited transcripts from the 133 metatranscriptomes. We report the maximum transcription for each MAG and denote the top 15 most transcriptionally dominant MAGs from each bacterial or archaeal domain (Fig. 1B and C). These MAGs with dominant transcription also have high transcriptional occupancy, with a minimum detection in 35% across the 133 metatranscriptome samples (Fig. 1C; Table S5). A majority (83%) of these transcriptionally dominant genomes belong to poorly characterized genera as noted by their names (e.g., genera defined only by alphanumeric code). The prevalence of transcriptionally dominant but poorly classified genomes reveals a gap in our understanding of the organisms actively shaping wetland biogeochemistry.

### Connecting transcribed biogeochemical functions to microbial taxonomy

Each of the 1,948 MAGs that recruited transcripts was assessed for expressed gene content relating to 14 biogeochemical processes: oxygen (aerobic respiration, microaerophilic respiration), methane (methanogenesis, aerobic methanotrophy, anaerobic methanotrophy), sulfur (sulfur oxidization, sulfur reduction), nitrogen (nitrification, nitrogen reducer, nitrogen fixation, and dissimilatory nitrate reduction to ammonium [DNRA]), iron (iron oxidation, iron reduction), and other metabolisms (phototrophy, obligate fermentation) (Fig. S1 and supplemental methods, see Methods). While MAG-based analyses are inherently constrained by completeness, manual curation and expression-based validation minimized bias associated with incomplete or contaminated bins. Our analyses resulted in 1,135 MAGs that contained sufficient gene transcript recruitment to be assigned to at least one of these 14 biogeochemical guilds (Table S6).

**Fig 2:**
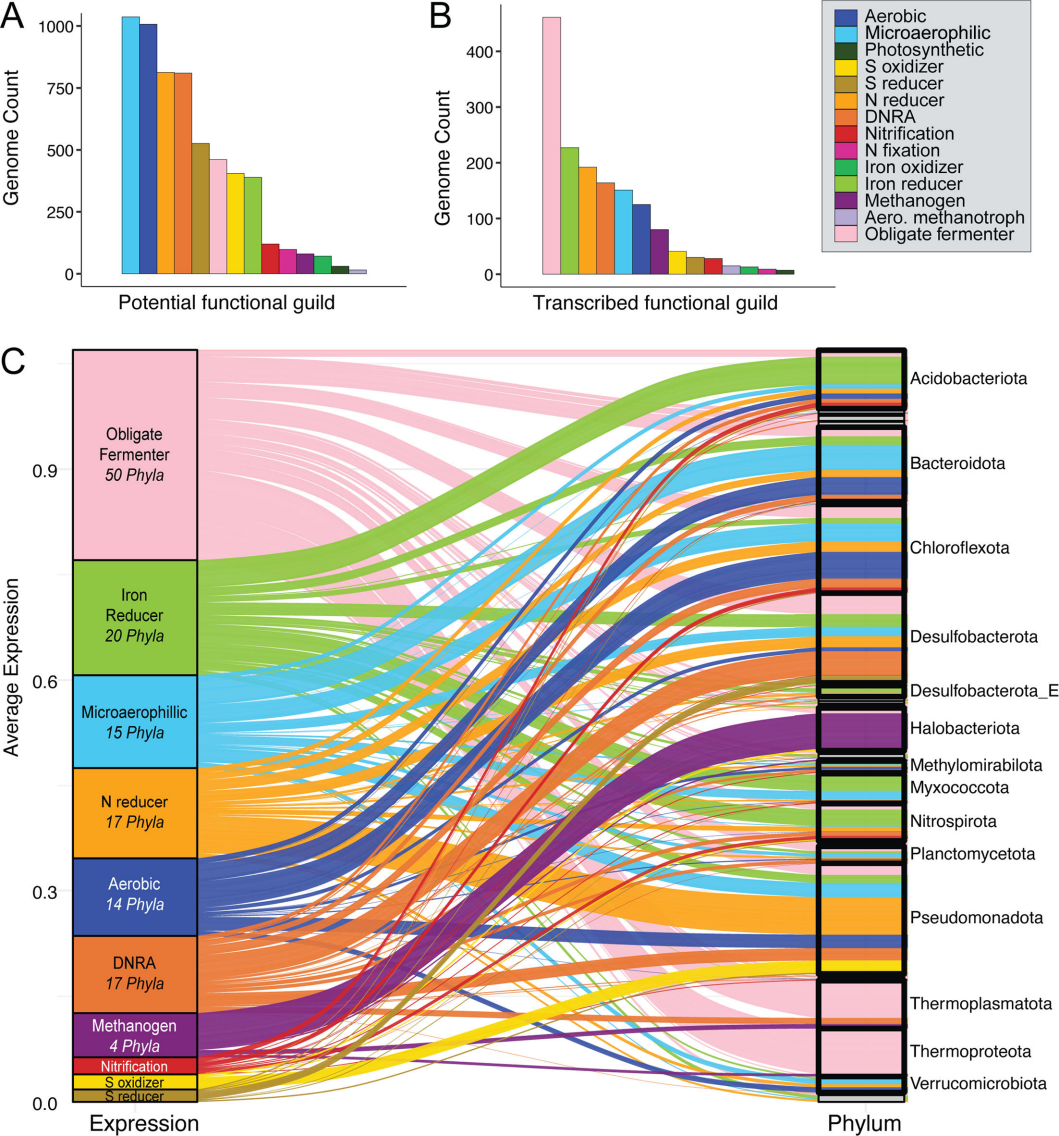
Distinct functional potential and expression across wetland microbial genomes**.** Bar plots summarize the genome counts across functional traits for genomic potential (**A**) and transcription (**B**), respectively. (**C**) Alluvial plot illustrates the distribution of top 10 expressed metabolic guilds (left) and their corresponding phyla (right). The height of each flow represents the average expression of that metabolic guild in a particular phylum, with colors indicating different metabolic guilds. Labels for phyla with less than 0.01 total expression were omitted. The number of phyla for each guild is noted for 7 of top 10 phyla, with nitrification (*n* = 11 phyla), S oxidizer (*n* = 4 phyla), and S reducer (*n* = 7 phyla) guilds detailed here.

Aerobic and microaerophilic metabolisms were the most widely encoded across wetland lineages (Fig. 2A). We compared the dominant encoded metabolic traits (i.e., those detected in the greatest number of genomes) to the most dominant transcribed traits, finding that the transcribed functionalities did not follow the encoded traits (Fig. 2B). For example, the capacity for microaerophilic and aerobic respiration was detected in the largest percentage of genomes (53% and 51%), but only detected in 8% and 6% of genomes in our transcriptomes, respectively. In contrast, transcription of genes for anaerobic metabolisms, such as obligate fermentation, iron reduction, and nitrogen reduction, was detected more frequently than genes for aerobic metabolisms. This enrichment for anaerobic metabolisms in the transcript signal is not entirely surprising given that most soils in our data set have porewater dissolved oxygen (DO) concentrations below the 1 µM detection limit (Table S4). Our approach reveals that transcriptional profiles capture environmentally responsive metabolic activity not evident from genome content alone, underscoring the importance of expression-based profiling to uncover active microbial processes.

Focusing on transcribed functions at the phylum level, we found that certain phyla, such as Thermoproteota or Halobacteriota, exhibited functional specialization, with transcription dominated by obligate fermentation or methanogenesis (Fig. 2C). In contrast, phyla such as the Pseudomonadota and Acidobacteriota transcribed a wide array of functional traits, reflecting substantial metabolic multifunctionality. This analysis also reveals disproportional contributions from phyla to nutrient and redox transformations. For instance, MAGs transcribing genes for sulfur reduction are enriched in the Desulfobacterota, iron reduction is prevalent in the Acidobacteriota and Nitrospirota, aerobic respiration is prevalent across the Chloroflexota, and nitrogen reduction and sulfur oxidation are enriched in some lineages of the Pseudomonadota. Notably, keystone processes such as nitrification and nitrogen fixation were both rare (<5% of MAGs) and phylogenetically dispersed (Table S6). Nitrogen fixation, for example, was detected in transcripts from just nine MAGs across three phyla, encompassing aerobes, iron reducers, and denitrifiers, as well as a methanogen and a nitrifier, illustrating how assimilatory and dissimilatory processes are coupled. This trait mapping reveals the phylogenetic distribution of key metabolic activities and underscores the diverse, active roles that microorganisms play in driving nutrient and redox transformations.

### Identifying the dominant drivers shaping wetland microbiome structure and function

**Fig 3:**
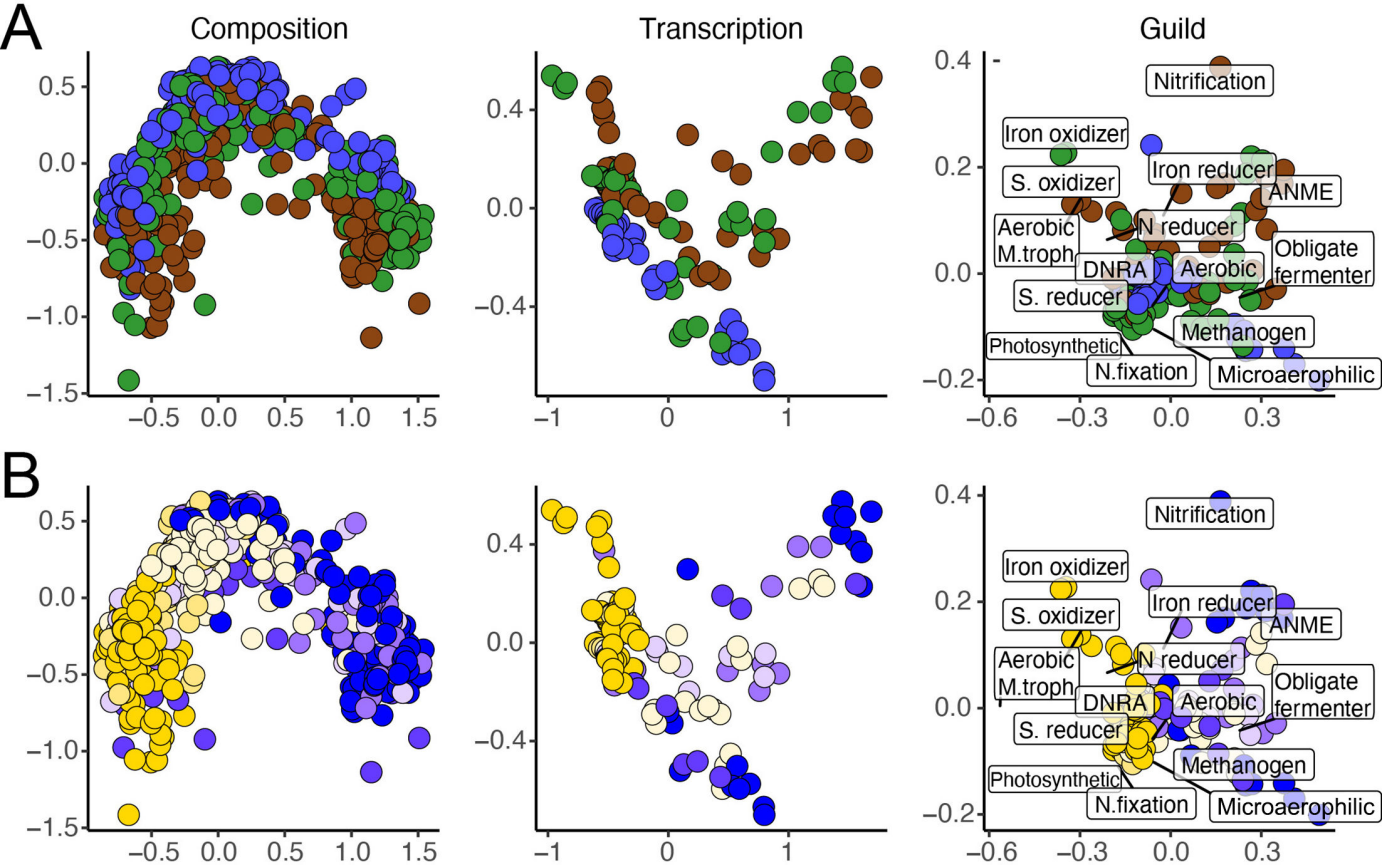
Depth structures wetland soil microbiomes. (**A**) Nonmetric multidimensional scaling (NMDS) ordinations of wetland microbiome structural metrics are organized from (left) overall composition (*N* = 671), (middle) genome transcription (*N* = 133), and (right) guild transcription (*N* = 133). Samples in panel **A** are colored by land cover (brown = mud; green = vegetative; blue = open water), while panel **B** is colored by depth (yellow = surface; blue = deep). Panels A and B highlight the responses of the two major environmental predictors, with depth being the strongest predictor across all soil microbiome attributes (Fig. S3).

To test how different wetland environments influenced the soil microbiome, we evaluated the impact of 17 spatial, temporal, and geochemical factors across three levels of biological organization: (i) phylotype membership, (ii) transcriptionally “active” genome membership, and (iii) expressed functional guilds (Fig. 3). Our analyses revealed that, regardless of the microbiome attribute measured, depth and depth-related geochemistry (such as soil Fe(II) and cation exchange capacity [CEC]) were the best predictors of wetland soil microbiome structure and function, more so than differences among land cover types (significance reported in Table S7). These chemical and physical soil attributes played a more direct role in shaping expressed functional guilds, explaining 40–50% of this variation, nearly double the variation explained for the phylotype data (Fig. S3). Overall, this information can inform wetland microbiome sampling priorities, showing the value of adding more depth-resolved measurements and affirming the collection of high-priority geochemical variables, such as CEC, Fe(II), pH, and acetate, while also recognizing that porewater oxygen measurements using handheld probes may fail to resolve redox microsite heterogeneity that drives wetland biogeochemical activity (6).

### Uncovering newly defined archaeal roles in carbon cycling

A valuable component of our sampling was the broad recovery of archaeal genomes (*n* = 298 MAGs), spanning 10 phyla with 48 families, including 88 genera. When considering the functional roles of Archaea in wetlands, these microorganisms were historically regarded primarily as methanogens or metabolizing other single carbon (C1) compounds (21, 29–31). Here, we derived a gene-based rule set to assign these genomes to carbon decomposition guilds of (i) polymer hydrolysis, (ii) sugar oxidation, (iii) organic acids and nitrogen conversions, and (iv) C1 metabolisms (Table S8).

**Fig 4:**
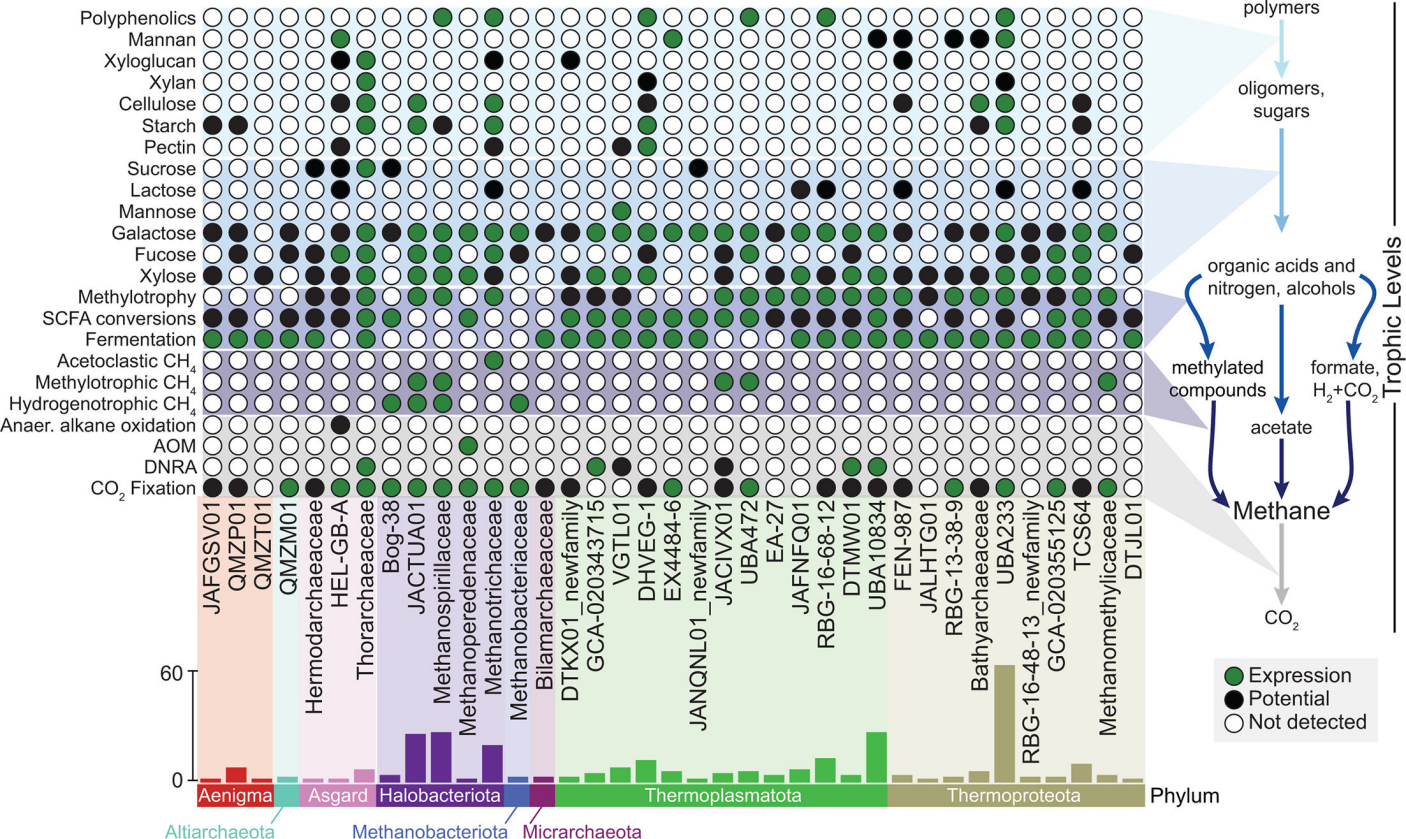
Assigning archaeal roles in the wetland carbon cycle. (Right) The schematic on the right visualizes the major trophic levels contributing to soil carbon decomposition, including light blue polymers (polyphenolics, mannan, xyloglucan, xylan, cellulose, starch, pectin); medium blue oligomers and sugars (sucrose, lactose, mannose, galactose, fucose, xylose); dark blue organic acids, nitrogen, and alcohols (fermentation, methylotrophy, anaerobic alkane oxidation); purple methanogenesis (acetoclastic, methylotrophic, and hydrogenotrophic); and gray other one-carbon metabolisms (anaerobic methane oxidation, CO_2_ fixation). (Left) For each archaeal family, the bubble plot denotes genes transcribed (green), genes present (black), or genes absent (white) for the major carbon utilization pathways assigned to the trophic levels. (Bottom) The bar chart summarizes the number of transcriptionally active MAGs recovered within each archaeal phylum and family.

We show phylogenetically diverse archaeal lineages expressed genes for hydrolyzing plant polymeric constituents, yielding sugars and other oligomers or monomers for the second trophic group (Fig. 4). Lineages such as Thorarchaeaceae and UBA233 transcribed genes to use six or more substrates that span both polymeric and sugar trophic groups. We also identified sugar-utilizing specialist archaea (e.g., members of the TCS64) that expressed genes for utilizing at least three sugar substrates. In support of these findings, expressed sugar metabolisms, including raffinose-, sucrose-, and maltose-like compounds, were prevalent across the metabolome data (Table S9 and S10), giving further credence to the likelihood of these metabolisms *in situ*. These findings provide evidence that Archaea, often overlooked in primary carbon degradation, contribute far more broadly to soil carbon turnover than previously recognized, occupying roles across multiple carbon trophic levels.

The third guild, prior to methane production, yields small-molecular-weight compounds such as organic acids (e.g., acetate, butyrate, lactate) and methylated (e.g., methylamine, methanol) compounds. Some of these are methanogenic substrates, while others can be further fermented, typically syntrophically, to yield methanogenic substrates. This latter guild had the greatest transcriptional prevalence across the archaeal genomes from seven phyla, including Aenigmatarchaeota, Altiarchaeota, Asgardarchaeota, Halobacteriota, Micrarchaeota, Thermoplasmatota, and Thermoproteota. This highlights the critical role these archaea, most of them classified as obligate fermenters, could play in cross-feeding methanogens.

The fourth guild of carbon is C1 metabolisms, with nearly a third of the archaeal genomes assigned as methanogens, representing four phyla (Methanobacteriota, Thermoproteota, Halobacteriota, Thermoplasmatota). The 85 methanogen MAGs spanned 10 families and 20 genera, eight of which were newly identified genera here (Fig. S4). Beyond canonical methanogens, we also recovered genomes from presumed non-methanogenic, *mcrA-*containing taxa such as the anaerobic methanotrophic *Methanoperedens* genus and the alkane-oxidizing Helarchaeales. Prior to this effort, the largest wetland MAG database contained 21 methanogen representatives with 4-fold less genus-level diversity (10). Despite extensive study of wetland methanogenesis, the recovery of additional active and previously undescribed methanogens from a single site underscores the continued expansion of genomic diversity in these systems.

Beyond taxonomic novelty, metabolic reconstruction with paired transcriptional profiles offered new insights into wetland methanogenesis (Fig. S4; Table S11). Acetoclastic methanogenesis was confined exclusively to *Methanothrix* and closely related members (16 MAGs). Our field data reinforce laboratory studies of *Methanothrix* and *Methanosarcina* (32), indicating that obligate acetoclastic *Methanothrix* outcompetes the facultative acetoclast *Methanosarcina in situ* due to more efficient substrate acquisition under the low acetate concentrations typical of these wetland soils (mean 45 µM; Fig. S5). Across the methanogens, obligate hydrogenotrophy was the most prevalently transcribed pathway in 52% of the MAGs, with two of the most transcriptionally active Archaea identified as obligate hydrogenotrophs (*Methanoregula* and UBA9949; Fig. 1). This work confirms and extends canonical methanogenic paradigms by showing not just potential, but active dominance of *Methanothrix* and *Methanoregula* under natural, low-substrate field conditions.

While wetland CH_4_ is commonly attributed to acetoclastic and hydrogenotrophic methanogens (3), our findings highlight the overlooked importance of methylotrophic pathways. We found that 25% of wetland methanogen MAGs expressed genes for methylotrophic methanogenesis, and one of the most transcriptionally active Archaea, *Methanomethylicus*, was a methylotroph (Fig. 1C; Table S5). Additionally, we detected methylotrophic gene expression in lineages traditionally considered hydrogenotrophic, including *Methanoregula* MAG and one MAG belonging to a novel genus within the *Methanospirillaceae* family (Fig. S4). We further resolved substrate preferences for methylotrophic methanogens, showing specificity for methylated nitrogen, sulfur, and oxygen compounds. Supporting this, NMR and LC-MS analyses frequently detected methyl-N compounds (e.g., betaine, carnitine) and methyl-O compounds (e.g., methanol, vanillic acid) at higher concentrations and in more samples than canonical substrates such as formate or acetate (Fig. S5). These findings reinforce an emerging consensus that methylotrophic methanogenesis may be a widespread and underappreciated source of methane in wetland ecosystems (21, 31, 33).

### Methane-cycling guilds are temporally stable along a depth gradient over years

Methane-cycling genome-based transcription patterns from a flooded mud flat collected monthly over a 3-month summer 2018 season exposed methanogen and methanotroph spatiotemporal niches and possible depth-defined ecotypes (Fig. S6). We uncovered six core methanogens, from diverse functional and phylogenetic backgrounds, with high levels of mean transcription across all soil depths and time points. The remaining CH_4_ cycling members were localized to specific soil depths, forming co-expressing clades that persisted across this summer season. We report a greater proportional enrichment of acetoclastic and aerobic methanotrophs in surface soils, while the middle and deepest soil layers included the anaerobic methane-oxidizing *Methanoperedens* genome and proportionally more hydrogenotrophic and methyl-utilizing methanogens. Reflecting these latter methanogens’ requirements for hydrogen, we observed increased formate concentrations at deeper (10–30 cm) depths across our mud, plant, and open water land cover patch types (Fig. S5). Our findings highlight the persistence of depth-stratified methane “neighborhoods,” composed of many closely related strains with a high degree of functional redundancy.

**Fig 5:**
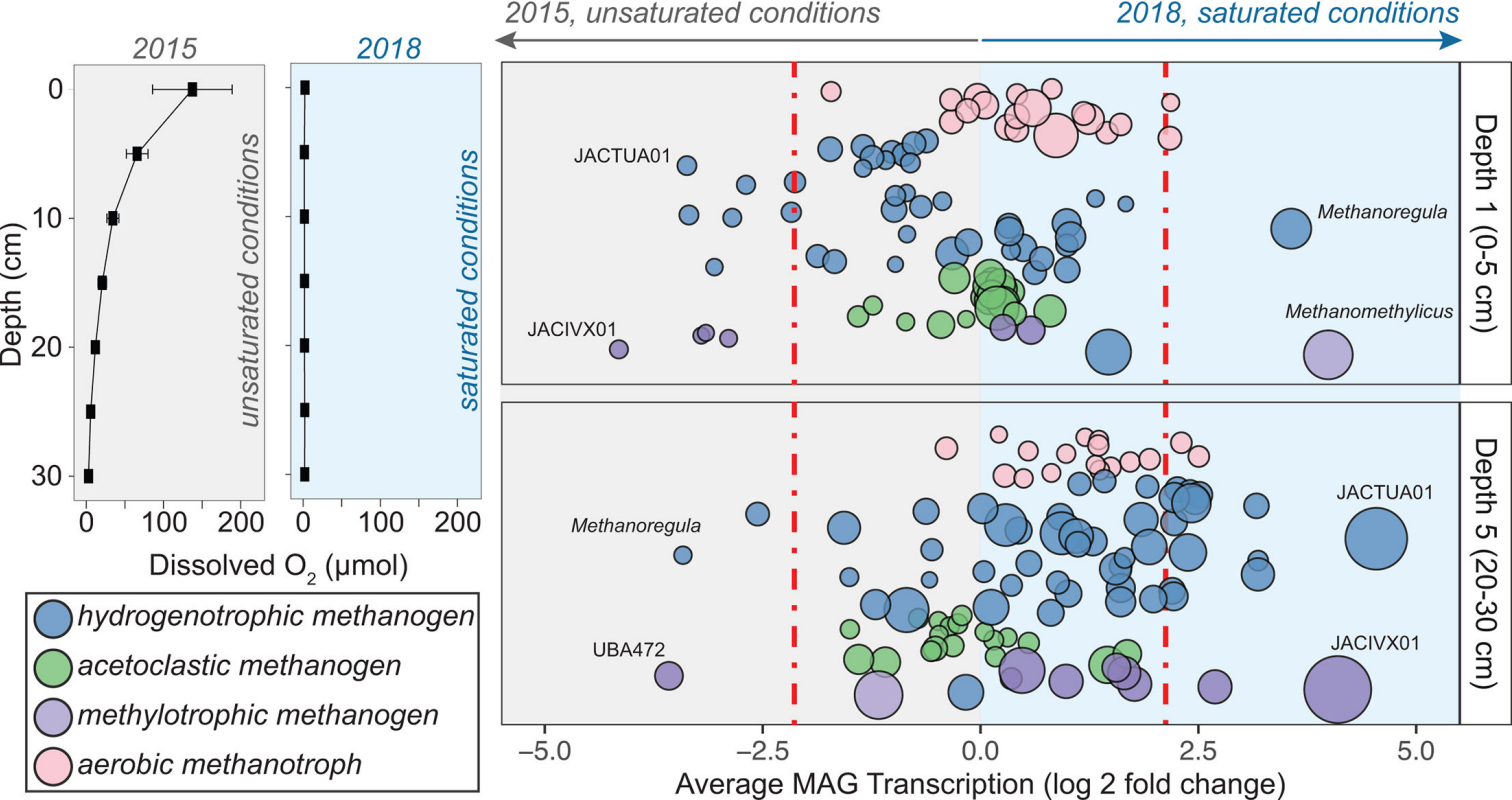
Methanogen stability in response to flooding. Dissolved porewater oxygen depth profiles from August 2015 (gray) and 2018 (blue) highlight the redox impact of flooding. The scatterplot shows the change in mean MAG transcription from 2015 to 2018 for the methane-cycling community within surface (0–5 cm) and deep (20–30 cm) soil compartments. Bubbles represent a single methane-cycling MAG and are colored by metabolism and sized by mean transcription. MAGs within the red dashed lines do not have log_2_ fold change in mean transcription and are considered temporally stable.

Given the seasonal stability across summer 2018, we next compared the multi-omics data collected from this site in August 2018 to parallel samples from the same mud flat in August 2015. During this time, the wetland flooded, and the exposed mud flat from 2015 was beneath 5.3 ft of overlying freshwater in 2018. Based on existing theory about the redox sensitivity of methanogens and their responses to hydrological perturbations (34), we hypothesized that flooding would decrease dissolved oxygen (DO) concentrations and increase methanogen expression broadly across the community, particularly in surface soils. Matching flooding expectations, DO decreased to non-detectable levels in surface soils subsequent to flooding. In contrast to expectations, 90% of the methanogen and aerobic methanotroph (Methylococcaceae, Methylomonadaceae) MAGs experienced no significant change in mean genome transcription in the flood-restructured surface soils (Fig. 5). Worth noting, a few taxa were flooding-responsive, such as the methylotrophic *Methanomethylicus* (Fig. 1C). Supporting this lack of restructuring in the CH_4_ cycling microbiome, the concentrations of methanogenic substrates, such as formate, methanol, and acetate, did not change with flooding (Fig. S5). This flooding response may be due to the fact that methanogens in these routinely submerged and drained surface soils could be already sheltered within anoxic microsites, such as biofilms or soil aggregates (6, 35).

### Co-expression networks reveal key lineages modulating soil greenhouse gases

**Fig 6:**
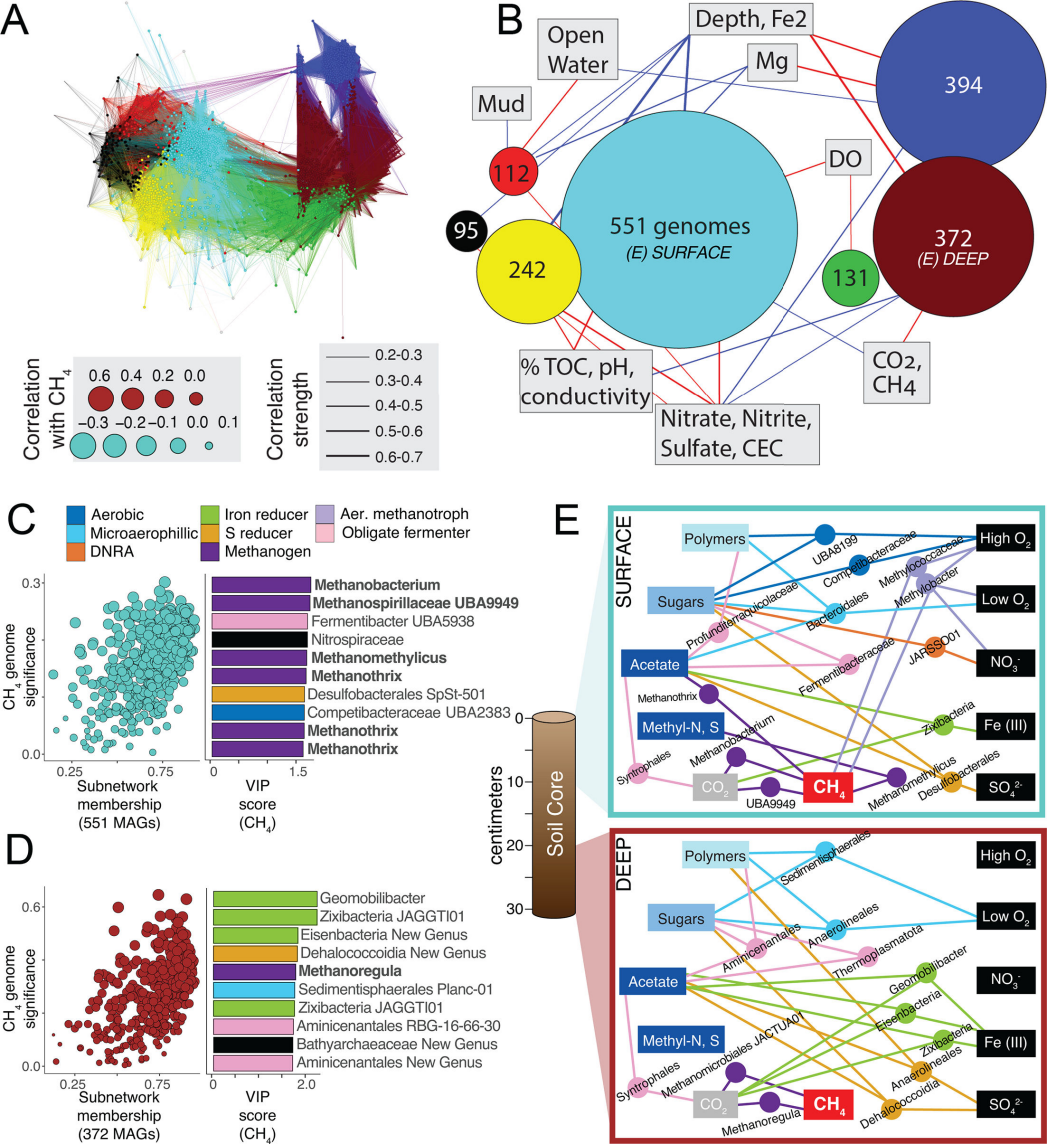
Coordinated gene expression networks predict wetland methane concentrations. (**A**) Overall co-expression network from 1,948 MAGs across 133 metatranscriptomics samples. Node colors denote distinct co-expression modules (*N* > 100 per module) identified using weighted gene co-expression network analysis (WGCNA) and are applied consistently throughout the figure. (**B**) A simplified network diagram highlighting the associations between modules (sized scaled by number of MAGs) and environmental features. Red and blue edges correspond to positive and negative associations, respectively. (**C and D**) Scatter plots represent two subnetworks, with points being MAGs, based on their correlation to CH_4_. (**C**) The genome significance plot and highest variable important genomes related to CH_4_ concentrations in the turquoise, surface-derived subnetwork (*n* = 551 MAGs, Rho = 0.53, *P* < 0.001). (**D**) The genome significance plot and highest variable important genomes related to CH_4_ concentrations in the brown, deep subnetwork (*n* = 372 MAGs, Rho = 0.58, *P* < 0.001). For panels C and D, the bar charts (middle) highlight the relative contribution of the top 10 variably important genomes (colored by metabolic classification). Methanogens are highlighted with bold text. (**E**) A process-based schematic based on genome-resolved transcript data of the significant variable important projection (VIP, >1) taxa and their contribution to methane production in the surface subnetwork from panel **A** (top) and the deep subnetwork from panel **B** (bottom).

We implemented a workflow to integrate the data collected across this manuscript to render the carbon and energy trophic network predictive of soil CH_4_ concentrations (Fig. S7). First, weighted gene co-expression identified seven modules composed of genomes with shared transcriptional patterns across our 133 metatranscriptome samples (Fig. 6A). Two of these modules predicted soil porewater CH_4_ concentrations, with the surface-soil–associated turquoise module negatively correlated (Rho = 0.53, *P* < 0.001) and the deeper-soil–associated brown module positively correlated (Rho = 0.58, *P* < 0.001) with soil CH_4_ concentrations (Fig. 6A and B; Table S12). These surface and deep modules contained 551 and 372 MAGs, respectively. The top 10 microbial genomes with the greatest importance for CH_4_ prediction and their transcribed biogeochemical traits are also noted (Fig. 6C and D). Distilling the transcribed carbon and energy functional gene content of these predictive genera, we mapped the trophic configurations that contribute to CH_4_ concentrations in these depth-defined compartments (Fig. 6E; Table S13).

Consistent with our taxonomic-based analyses, we found that key biogeochemical processes (such as iron reduction, microaerophilic respiration, and methane production) were transcribed across depths in these predictive modules (Fig. 6C and D). The iron-reducing Zikibacteria and the obligate fermenter Syntrophales were important predictors in both depth compartments. However, distinct microbial lineages were often responsible for mediating the same functions at different depths. For instance, microaerophilic respiration involved *Bacteroidales* in surface soils, while *Anaerolineales* and *Sedimentisphaerales* fulfilled similar roles in deeper soils. The turquoise surface module also included functions not observed in the deep module, such as nitrate reduction and low-affinity cytochrome oxidase expression, traits adapted to higher oxygen conditions. Additionally, transcriptional evidence for aerobic methanotrophy (*Methylococcaceae* UBA6136 and *Methylomonadaceae* KS41) was exclusive to the surface module. This likely explains the negative correlation between the turquoise module and CH_₄_ concentrations in surface soils (Fig. 6B).

Together, these transcriptome-derived, process-based observations reveal expressed biogeochemical interactions in wetland soils. Soil CH_₄_ concentrations appear to be emergent properties of the microbiome, driven not by individual methanogens alone, but by complex, depth-resolved interactions among microbial guilds. Notably, the strongest predictors of soil porewater CH_₄_ concentrations were often upstream carbon-oxidizing organisms such as iron reducers (*Zikibacteria, Eisenbacteria, Geomobilibacter*) and fermenters (*Aminicenantales*) that likely support diverse functional methanogens (*Methanoregula, Methanothrix, Methanomethylicus*). While soil Fe(II) concentrations were strong drivers of microbiome structure, genome-resolved transcriptional data provide organism-level resolution, identifying specific iron-reducing taxa co-occurring with methanogens and clarifying potential interactions underlying depth-dependent redox processes. These findings underscore the need for higher-throughput tools to resolve microbial interactions in the context of ecosystem-scale methane dynamics.

## DISCUSSION

### Depth-structured microbiomes and redundancy shape wetland stability

Our study shows that depth is the dominant axis structuring microbial composition and transcriptional activity in freshwater wetland soils. Across spatial and temporal gradients, microbial communities and biogeochemical functions were vertically stratified, with functional guilds consistently aligned with depth-defined redox and geochemical profiles. Traits such as methanogenesis, iron reduction, and fermentation were expressed by phylogenetically diverse organisms within the same depth layers, indicating high transcriptional redundancy. This redundancy, both within and across lineages, likely buffers methane-cycling functions against environmental fluctuations and contributes to the long-term stability of CH_₄_ production. While previous inferences of functional redundancy were drawn from alpha diversity or genome-level data (36), our findings demonstrate this redundancy at the level of gene expression and functional traits, offering mechanistic evidence of activity-based ecosystem resilience.

Rather than interpreting these results as a comprehensive census of total microbial diversity, we observed widespread transcriptional activity across a large portion of recovered archaeal and bacterial genomes. Specifically, 78% of the 2,502 genomes recruited transcripts, with 60% of these MAGs expressing sufficient genome content to permit metabolic assignments to 14 traits associated with wetland biogeochemistry. Moreover, the average MAG mean transcriptional occupancy was 59%, signifying that active members were maintained across samples spanning diverse wetland gradients. While genome-resolved approaches do not capture the full diversity of a system, our findings demonstrate that a substantial portion of the active wetland microbiome is maintained across depth and time. This widespread activity, combined with high functional redundancy, likely contributes to ecosystem stability but also complicates methane mitigation. In highly redundant systems, inhibiting one lineage or process may simply lead to functional compensation by others (37–39), a microbial game of “whack-a-mole.” As such, successful mitigation strategies must move beyond single-target interventions and account for the depth-resolved, community-level architecture of soil microbiomes.

### Expanding the methane paradigm: beyond classical substrates and lineages

This study also expands the framework of methane cycling by identifying alternative metabolic routes and underappreciated contributors. Although acetoclastic and hydrogenotrophic methanogenesis dominate conventional models (40), nearly a quarter of transcriptionally active methanogens in our study expressed genes for methylated compound utilization. These findings suggest that CH_₄_ production is not just fueled by acetate or hydrogen but also by methylated nitrogen-, sulfur-, and oxygen-containing substrates. Moreover, methane concentrations in porewater were more accurately predicted by co-expression networks involving iron reducers, obligate fermenters, methane oxidizers, and microaerophilic heterotrophs than by methanogens alone. These data reveal methane as an emergent property of integrated, depth-specific microbial consortia. In surface soils, aerobic methanotrophs likely attenuate CH_₄_ as it is produced (based on the strong spatial co-expression), while deeper modules are dominated by organisms facilitating substrate production and syntrophic hydrogen production (Fig. 6).

These findings reinforce the need for methane mitigation strategies that are not only taxon-specific but also spatially and functionally informed. Interventions that ignore the trophic structure and spatial organization of microbial communities risk being ineffective or only short-lived. For example, in other methanogenic systems such as the rumen (41, 42), mitigation approaches increasingly target upstream methanogen metabolic partners, manipulate redox conditions, or reroute electron flow to non-methanogenic microbes. Similarly, recent work has shown that catechin, a naturally occurring plant metabolite found in wetland soil microbiomes, can suppress methane production by up to 84% by redirecting hydrogen flux away from methanogens (38). Especially in complex environments like saturated soils, precision strategies that consider microbial interdependencies, metabolic plasticity, and depth-resolved activity are likely to offer more robust and scalable outcomes for methane mitigation.

### Implications for redox-informed Earth system models

Our genome-resolved transcriptional profiles reveal seasonal and multi-year stability in methane-cycling activity across soil depths, despite substantial hydrological and redox perturbations. While we previously reported elevated CH_₄_ production under oxygenated conditions at this site in 2015 (6, 15), new flooding data reinforce that methanogen transcription is largely unresponsive to shifts in soil oxygen concentrations. These findings add to a growing body of evidence challenging the long-standing paradigm that oxygen is the primary regulator of wetland CH_₄_ production (31–34). In our data, methanogen transcripts persisted in surface soils with detectable oxygen and showed no significant change following a 5.3-foot, flood-induced redox shift, while aerobic methanotrophs remained active even under anoxic conditions. These patterns likely reflect fine-scale redox heterogeneity, including anoxic microsites, variable oxygen exposure, or metabolic flexibility that enables persistence under low-oxygen conditions. Our results expose the limitations of using coarse redox proxies, such as dissolved oxygen, in Earth system models and underscore the need for finer-scale, spatially explicit observations. Incorporating more tailored biogeochemical measurements and genome-informed proxies would improve representation of methane-cycling heterogeneity in wetlands.

Current biogeochemical models also largely neglect methylotrophic methanogenesis, despite mounting evidence that transcriptionally active methanogens frequently utilize methylated nitrogen-, sulfur-, and oxygen-containing compounds (21, 31, 43–45). Metabolomic profiling further revealed a diverse pool of methylated compounds, including potential and previously underappreciated methanogenic substrates, supporting the ecological relevance of methylotrophic pathways inferred from transcriptional data. Importantly, many of these potential but unknown substrates, such as trimethyllysine, are structural homologs of compounds known to be utilized by methanogens (or physiologically analogous methylotrophic acetogens), supporting their possible conversion to methane. Methylotrophic organisms have increasingly been shown to utilize diverse substrates; therefore, these diverse methylated compounds, which we show are abundant *in situ*, may represent a substantial and previously under-accounted source of CH_₄_ emissions. Together, these findings highlight the need for additional targeted studies to identify, quantify, and evaluate the role of specific methylated substrates contributing to methane production in wetland systems.

To incorporate these pathways into predictive frameworks, targeted cultivation of methyl-reducing methanogens is needed (44, 46), particularly from wetland environments where cultured representatives remain scarce. Such efforts are essential for generating key physiological parameters (e.g., uptake kinetics, growth optima, yields), paralleling existing knowledge for acetate- and hydrogen-utilizing methanogens. In parallel, stable isotope-based approaches will be vital for tracing substrate fate and quantifying the relative contribution of methylotrophy to CH_₄_ emissions. To help focus these efforts, our transcriptomic analysis distilled 85 methanogens into a “most wanted” target list of four genera (*Methanoregula*, *Methanothrix*, *Methanomethylicus*, and Methanospirillaceae UBA9949) to be prioritized for cultivation based on their transcriptional activity and predictive value for CH_₄_ concentrations (Fig. 1 and Fig. 6). Supporting this prioritization, a recent cross-site comparison across nine temperate wetlands (33), including Old Woman Creek, found *Methanoregula* and *Methanothrix* to be core taxa, with *Methanoregula* consistently emerging as a network hub strongly predictive of CH_₄_ flux. As methane remains a powerful lever for near-term climate mitigation (39), advancing the biochemical and ecological understanding of these keystone methanogens and the environmental controls shaping their activity will improve how models represent methane production pathways and reduce uncertainty in predicted wetland CH_₄_ emissions.

Accurately predicting methane flux in wetlands requires a shift toward models that capture the functional and spatial organization of microbial communities. Recent advances, such as the genome-to-ecosystem framework, have shown that incorporating genome-inferred biogeochemical traits weighted by community composition can markedly improve methane predictions in peatlands, reducing model bias by more than 50% (47). While impactful, these models rely solely on genomic potential, which our findings suggest may fail to capture the biogeochemical pathways actively driving emissions. Our work builds on and extends this approach by integrating metatranscriptomic data to define trophic modules that reveal depth-stratified networks of carbon oxidizers, syntrophic cross-feeders, terminal methanogens, and methane oxidizers. By identifying the most active and predictive processes, our findings provide a foundation for developing *in situ* diagnostics that move beyond multi-omic sampling toward cost-effective proxies of microbial function such as targeted monitoring of key marker genes, expression assays for predictive pathways, or geochemical indicators. Embedding activity-based functional traits into Earth system models will be critical for improving methane predictions and informing scalable, precision-guided interventions across diverse wetland ecosystems.

### Conclusion

In this study, we employed a comprehensive, multi-omics survey of wetland soils to generate an unprecedented view of the structural and functional microbiome attributes that underpin biogeochemical cycling. This approach helps move soil microbiome research beyond the historical “black box” of environmental inputs and outputs, revealing how microbial community composition and gene expression respond to depth and geochemical gradients. To interpret this complexity, we developed gene-based rules to classify microbial energy metabolisms and carbon trophic guilds, enabling us to resolve emergent properties of methane-cycling consortia. This framework uncovered previously unrecognized roles for archaeal lineages in carbon decomposition, revealed the substrate versatility and redox resilience of methanogens, and demonstrated stable, depth-stratified co-expression patterns predictive of soil CH_4_ concentrations. Together, these insights provide a mechanistic foundation for embedding microbial activity into Earth system models and for guiding microbiome-informed strategies to mitigate methane emissions from wetlands.

## MATERIALS AND METHODS

See Supplemental Information for detailed materials and methods, including field sampling design, geochemical and metabolomic analyses, 16S rRNA gene sequencing, metagenomic and metatranscriptomic sequencing, genome assembly and binning, trait assignment workflows, and statistical and network-based analyses linking microbial activity to methane dynamics.

## Data Availability

The metagenomic reads, metatranscriptomic reads, bacterial and archaeal MAGs, and 16S rRNA gene sequencing reads reported here have been deposited in the National Center for Biotechnology Information (Table S1). To facilitate data accessibility, the 2,502 MAGs contained in this data set, identified here as MUCC (Multi-omics to understand Climate Change) v.1, are publicly available as a KBase data collection, offering genomes and annotations for use in an accessible, cyberinfrastructure-powered platform with the intent to advance wetland science broadly (https://narrative.kbase.us/narrative/147022). Flux and meteorological observations for OWC are available through Ameriflux, Site ID US-OWC (24). Porewater methane concentrations and chamber fluxes are available through ESS-DiVE (23, 48). Data for nutrient and carbon sequestration and soil accretion rates of OWC are available through ESS-DiVE (49). Additional meteorological, hydrological, and water quality data for OWC are available through the NERR data system (https://cdmo.baruch.sc.edu/). LC-MS data are available through MassIVE under accession MSV000093935. Additionally, all MAGs, derived gene calls, annotations, phylogenetic trees, and metatranscriptome abundance profiles are available on Zenodo under DOI 10.5281/zenodo.8194032.

## SUPPLEMENTAL MATERIAL

Supplemental Information msystems.00680-25-s0001.pdf Supplemental methods, figures, and table captions.

Supplemental Tables msystems.00680-25-s0002.xlsx Tables S1-S13.

ASM does not own the copyrights to Supplemental Material that may be linked to, or accessed through, an article. The authors have granted ASM a non-exclusive, world-wide license to publish the Supplemental Material files. Please contact the corresponding author directly for reuse.
