# Supplementary material for: Mapping the soil microbiome functions shaping wetland methane emissions
Source: mSystems. 2026 Jun 1;11(6):e00680-25. doi: 10.1128/msystems.00680-25 (PMC13289110; doi:10.1128/msystems.00680-25)
Supplement: Supplemental Information — Supplemental methods, figures, and table captions. [file msystems.00680-25-s0001.pdf]

Title: Mapping the soil microbiome functions shaping wetland methane emissions

Running title: Soil microbiome drivers of wetland methane emissions

Authors: Mikayla A. Borton[a+] (0000-0001-8037-4253), Angela M. Oliverio[b,c+] (0000-0002-0261-0393), Adrienne B. Narrowe[b] (0000-0002-2000-088X), Jorge A. Villa[d] (0000-0003-1130-9401), Christian Rinke[e] (0000-0003-4632-1187), David W. Hoyt[f] (0000-0002-2857-719X), Pengfei Liu[b,g] (0000-0003-1003-2025), Bridget B. McGivern[b] (0000-0001-9023-0018), Emily K. Bechtold[b] (0000-0001-5092-6264), Jared B. Ellenbogen[b] (0000-0003-1692-9044), Rebecca A. Daly[b] (0000-0003-2223-7458), Garrett J. Smith[h] (0000-0002-1014-5102), Jordan C. Angle[h] (0000-0002-3260-5851), Rory M. Flynn[b] (0000-0003-0709-2705), Andrew P. Freiburger[i] (0000-0002-7288-535X), Katherine B. Louie[j,k] (0000-0002-6787-7558), Brooke Stemple[b], Trent R. Northen[j,k] (0000-0001-8404-3259), Christopher Henry[i] (0000-0001-8058-9123), Christopher S. Miller[l] (0000-0002-9448-8144), Timothy H. Morin[m] (0000-0002-6504-6937), Gil Bohrer[n] (0000-0002-9209-9540), Kelly C. Wrighton[b#] (0000-0003-0434-4217)

[a] Department of Food Science and Human Nutrition, Colorado State University, Fort Collins, CO, 80521
[b] Department of Soil and Crop Sciences, Colorado State University, Fort Collins, CO, 80521
[c] Department of Biology, Syracuse University, Syracuse, NY, 13210
[d] School of Geosciences, University of Louisiana at Lafayette, Lafayette, LA 70503
[e] Department of Microbiology, University of Innsbruck, Innsbruck, Austria
[f] Environmental Molecular Sciences Laboratory, Pacific Northwest National Laboratory, Richland, WA, 99352
[g] Center for Pan-third Pole Environment, Lanzhou University, Lanzhou 730000, China
[h] Department of Microbiology, The Ohio State University, Columbus, OH 43210
[i] Data Science and Learning, Argonne National Laboratory, Lemont, IL 60439
[j] Lawrence Berkeley National Lab, Berkeley, CA, 94720
[k] DOE Joint Genome Institute, Lawrence Berkeley National Laboratory, Berkeley, CA, 94720
[l] Department of Integrative Biology, University of Colorado Denver, Denver, CO, 80217
[m] Department of Environmental Resources Engineering, State University of New York College of Environmental Sciences and Forestry, Syracuse, New York, 13210
[n] Department of Civil, Environmental & Geodetic Engineering, The Ohio State University, Columbus, OH, 43210

[+]**Authors contributed equally to this work:** Mikayla A. Borton and Angela M. Oliverio
[#]**Correspondence:** wrighton@colostate.edu
Keywords: genome, MAG, metagenomics, metatranscriptomics, global change, greenhouse gases, methanogen

This PDF file includes:

Methods
Supplementary Figures S1 to S7
Supplementary Data Tables S1 to S13
Supplemental References
Information on Zenodo content

Methods

Field campaign and sample collection. We extensively sampled the main wetland ecological sites (ecosites), or ecohydrological patch types, across two major field campaigns to capture the patch-level spatiotemporal heterogeneity. The patches included wetland plant cover (plant), predominantly *Typha latifolia*, exposed mud -flat (mud), and standing freshwater (open water) (**Fig. 1A**). Our sampling was also depth-resolved every 5 cm from 0-25 cm, resulting in a total of 705 soils included in this study (**Supplementary Fig. S1**).

Sampling was conducted at the Old Woman Creek National Estuarine Research Reserve (OWC). OWC is a freshwater wetland located on the southern shore of Lake Erie, OH, USA (41˚22'N, 82˚30'W). Samples were collected during field campaigns in 2013, 2014, 2015 and 2018. Sample collection details for the 2013-2014 and 2014-2015 campaigns were previously described (1–3). Consistent collection methods were employed for the 2018 campaign, which is first reported in this publication. Briefly, in 2018 we selected 10 sampling sites that include locations sampled during prior years and represent a range of current and historic patch types of Typha (n=1), Nelumbo (n=3), Open Water Channel (n=3), and Temporal Mud flat (n=3) (**Fig. 1A**). At each location, cores were collected within a meter of each of the 10 installed pore-water dialysis peepers (n=1 core for May, June, and n=3 cores for July, August, and September, during peak methane production at the wetland). Prior to core collection, we measured soil dissolved oxygen (DO) and temperature within 5 cm increments from the soil surface using the Fibox 4 meter and DP-Pst3 probe (PreSens Precision Sensing, Germany). Cores were hydraulically extruded and immediately divided into 5 cm depth sections from 0-25 cm, as measured from the core surface. Each 5 cm section was further subsampled for molecular and geochemical analyses: subsamples were flash frozen in the field and then shipped on dry ice for storage at -80˚C prior to RNA extraction.

New metagenomes generated in this study were selected based on prior 16S rRNA gene community profiles and methanogen genome recovery to prioritize samples representing key gradients in methane-cycling community structure and function. New metatranscriptomes were selected to capture temporal (July, August, September), vertical (D1, D3, D5), and spatial variability, including full-depth resolution in August and within-site replication across three cores at the mud site. New metabolomics samples were selected to pair directly with metatranscriptomes in order to link transcriptional activity to depth- and month-resolved metabolite dynamics.

Geochemical analysis and collection of gasses. Concentrations of soil nitrate, nitrite, acetate, and sulfate were measured using ion chromatography as previously described (1). Briefly, 1:1 soil slurries were made using 5g of soil with 5 ml MilliQ water. Following pH measurement, slurries were filtered using a 0.2µM filter, with the filtrate quantified on a Dionex ICS-2100 Ion Chromatography System with an AS18 column. Using soil slurry filtrate, concentrations were measured for: formate, methanol, acetate, at the Pacific Northwest National Laboratory using proton magnetic resonance (^{1}H NMR) as previously described (4, 5). Fe^{2+} was measured in the field using Hach FerroVer packets per the manufacturer's directions (Hach #927-9). Surface emissions of CH_4 and CO_2 were measured by non-steady-state chambers in duplicate in each ecosite and season and in sync with peeper measurement times (1). We also measured *in situ* concentrations of dissolved CH_4 and CO_2 belowground monthly via porewater dialysis samplers (1). In brief, peepers with a vertical resolution of 2.8 cm measured concentrations via a 0.1 µm membrane, allowing water inside the peeper windows to equilibrate with dissolved gas concentrations, with minimal soil disturbance. Gas concentrations from peepers were quantified with a Shimadzu GC-2014 gas analyzer as previously described (1). Data from these analyses are included in **Supplementary Table S4**.

Soil metabolomics methods. Water-soluble metabolites were extracted from soil by adding 7 mL of autoclaved Milli-Q water to 1 g of wet soil in a sterile 15-mL centrifuge tube. To identify field-present metabolites in Old Woman Creek, including methanogenic substrates, we performed 1H NMR on aliquots

water extractions. Samples (180 µL) were combined with 2,2-dimethyl-2-silapentane-5-sulfonate-d_6 (DSS-d_6) in D_2O (20 µL, 5 mM) and thoroughly mixed prior to transfer to 3 mm NMR tubes. NMR spectra were acquired on a Bruker Avance III spectrometer operating at a field strength of 17.6 T (^{1}H ν_0 of 750.24 MHz) and equipped with a 5mm Bruker TCI/CP HCN (inverse) cryoprobe with Z-gradient and at a regulated temperature of 298 K. The 90° ^{1}H pulse was calibrated prior to the measurement of each sample. The one-dimensional ^{1}H spectra were acquired using a nuclear Overhauser effect spectroscopy (noesypr1d) pulse sequence with a spectral width of 12 ppm and 1024 transients. The NOESY mixing time was 100ms and the acquisition time was 4s followed by a relaxation delay of 1.5 s during which presaturation of the water signal was applied. Time domain free induction decays (72114 total points) were zero filled to 131072 total points prior to Fourier transform. Chemical shifts were referenced to the ^{1}H methyl signal in DSS-d_6 at 0 ppm. The 1D ^{1}H spectra were manually processed, assigned metabolite identifications and quantified using Chenomx NMR Suite 8.6. Metabolite identification was based on matching the chemical shift, J-coupling and intensity of experimental signals to compound signals in the Chenomx and custom in-house databases**.** Quantification was based on fitted metabolite signals relative to the internal standard (DSS-d_6). Signal to noise ratios (S/N) were measured using MestReNova 14 with the limit of quantification equal to a S/N of 10 and the limit of detection equal to a S/N of 3. Data from these analyses are included in **Supplementary Tables S4 and S9**.

In preparation for LC-MS, water extracts were frozen and lyophilized dry (FreeZone 2.5 Plus, Labconco). Each extract was resuspended in 170 µL of 100% methanol containing isotopically labeled internal standards (5-50 µM of 13C,15N Cell Free Amino Acid Mixture, #767964, Sigma; 1 ug/mL 2-amino-3-bromo-5-methylbenzoic acid, ABMBA, #R435902, Sigma) and centrifuge-filtered (0.22 µm hydrophilic PVDF membrane, #UFC30GV00, Millipore) before transferring to glass LC-MS autosampler vials. To detect polar metabolites using LC-MS/MS, normal phase chromatography was performed using a QExactive HF Orbitrap MS (Thermo Scientific, San Jose, CA) in line with an Agilent 1290 LC stack. Chromatography was performed using a HILIC column (Agilent InfinityLab Poroshell 120 HILIC-Z, 2.1 x 150 mm, 2.7 µm, #673775-924) held at 40 ºC and mobile phase running at a flow rate of 0.45 mL/minute with 2 µL injection volumes for each sample. The column was first equilibrated with 100% buffer B (95:5 ACN:H2O with 5 mM ammonium acetate) for 1 minute, then diluting this down to 89% with buffer A (100% H2O with 5 mM ammonium acetate and 5 µM methylenediphosphonic acid) over 10 minutes, then down to 70% B over 4.75 minutes, then down to 20% B over 0.5 minutes, followed by isocratic elution in 80% buffer A for 2.25 minutes followed by re-equilibrating the column at 100% B prior to the next injection. Mass spectrometry data was collected in centroid mode in both positive and negative polarity and included full MS spectra from m/z 70-1050 at 60,000 resolution as well as MS2 fragmentation spectra acquired using stepped then averaged 10, 20 and 40 eV collision energies at 17,500 resolution. Source settings included spray voltage of 3kV for both positive and negative ionization, sheath gas flow of 55 (au), auxiliary gas flow of 20 (au), sweep gas flow of 2 (au), and capillary temperature of 400 degrees C. Sample injection order was randomized, with an injection blank of 100% methanol run between each sample and replaced by an internal standard or quality control mixture only every 3 to 15 samples. Metabolomics data is available through MassIVE under accession MSV000093935.

Metabolites were identified by comparing exact mass, retention time (RT) and MS/MS fragmentation spectra to that of compound standards run using the same chromatography and mass spectrometry methods. LC-MS spectra were analyzed using custom Python code (Yao et al., 2015), with a score of 0 to 3 assigned to each feature (unique m/z and RT pair) that represented the level of confidence in identifying the metabolite. A metabolite positively identified had a detected m/z of ≤ 5 ppm or 0.001 Da from theoretical and RT ≤ 0.5 minutes compared to that of a pure standard run using the same LC-MS conditions. Highest level of positive identification (score of 3) was given for features having matching fragmentation spectra to an internal database of standards run and collected on a Q Exactive Orbitrap MS,

with mismatching spectra invalidating a positive identification. Methodological processing these analyses are included in **Supplementary Tables S9-S10**.

DNA and RNA extraction. DNA was extracted from soil samples using the PowerSoil DNA kit (Qiagen). Total RNA was extracted using the Takara NucleoBond RNA Soil kit (Takara Bio USA, CA) with 5g of soil as input. Extracted RNA was purified and concentrated using the Zymo RNA Clean & Concentrator Kit (Zymo Research) and quantified with the Qubit RNA HS Assay (Thermo Fisher Scientific). For low biomass samples, up to 5 RNA extractions of 5g were pooled prior to concentration. RNA purity and DNA removal were verified by PCR using universal V4 region 16S rRNA gene primers.

16S rRNA gene amplicon sequencing. 16S rRNA genes in extracted DNA were amplified with the primers 515F/806R (6) and sequenced at Argonne National Laboratory on the Illumina MiSeq with 251-bp paired end read. Read sets (n=701) were processed with QIIME2 (release 2018.11, (7)) and ASVs were recovered with DADA2 (8). Taxonomies were assigned with the q2-feature-classifier with the SILVA database (release 132) (9). ASVs were only included in downstream analyses if they were detected in at least 10% of samples. We set a minimum read threshold of at least 5,000 sequences per sample, retaining 672 samples, and then normalized our ASV table with cumulative sum scaling prior to downstream analyses (**Supplementary Table S2**).

Metagenomic and metatranscriptomic sequencing. Of the 42 Metagenomes, 25 are newly reported data here (**Supplementary Table S1**). This includes 16 samples from the 2018 field sampling representing a range of patch types, soil depths and time points to be used in building a MAG database. For the 2018 field samples sequencing was performed by the Department of Energy Joint Genome Institute following their established protocols. Briefly, plate-based DNA library preparation for Illumina sequencing was performed on the PerkinElmer Sciclone NGS robotic liquid handling system using Kapa Biosystems library preparation kit. 200.20 ng of sample DNA was sheared to 472 bp using a Covaris LE220 focused-ultrasonicator. The sheared DNA fragments were size selected by double-SPRI and then the selected fragments were end-repaired, A-tailed, and ligated with Illumina compatible sequencing adaptors from IDT containing a unique molecular index barcode for each sample library. The prepared libraries were quantified using KAPA Biosystems' next-generation sequencing library qPCR kit and run on a Roche LightCycler 480 real-time PCR instrument. Sequencing of the flowcell was performed on the Illumina NovaSeq sequencer using NovaSeq XP V1 reagent kits, S4 flowcell, following a 2x151 indexed run recipe.

We also sequenced 9 samples from laboratory enrichments that are released in this study. Anaerobic enrichment experiments were conducted to enhance methanogen and methanotroph genome recovery, with the resulting metagenomes and metagenome bins reported here. Wetland sediment was collected from M1 site at 20-30cm depth in August 2018, and approximately 10 g of material was transferred into serum bottles containing basal anaerobic medium amended with coenzyme M (10 mg L^{-1}). Bottles were prepared under an N_2/CO_2 atmosphere and incubated in the dark at 25 °C. Enrichments were amended with individual substrates, including methanol (3 mM), trimethylamine (TMA; 1 mM), pectin (500 mg L^{-1}), acetate (200 mM), 3,4,5-trimethoxybenzoic acid (TM345B; 10 mM), or casamino acids (0.1%), with selected treatments additionally supplemented with 10% H_2 in the headspace. Control incubations without added substrate were included. Substrates were added monthly for 4 months, with headspaces flushed with N_2/CO_2 between amendments to maintain anaerobic conditions. A subset of enrichment cultures were sampled for metagenomic sequencing after 4 months.

Two of these enrichment samples were sequenced at The Ohio State University Genomic Shared Resource Facility with library preparation Nextera XT library system and sequencing done on HiSeq 2500 using methods described in more detail (10). An additional 7 laboratory enrichments were sequenced in 2018 from Genomics and Microarray Shared Resource at University of Colorado Denver

Cancer Center, Denver, CO, USA. For these samples, library preparation was done (via Tecan/Nugen Ovation Ultralow DNA Input Kit, No. 0344) and sequenced on the Illumina NovaSeq 6000, with an S4 flow cell. These enrichments were used to try to enhance methanogen recovery, with the resulting metagenomes and metagenome bins reported here. While these enrichments are not the focus of this study, methods for enrichment generation are outlined above.

We obtained 133 samples from our metatranscriptomics sequencing across the multi-year study, with their distribution across land coverages, sites, cores, years, months, and depths reported (**Supplementary Table S1, S4**). From the field in 2018 we obtained 109 metatransciptome samples from triplicate cores for each of 3 depths (0-5 cm, 10-15 cm, and 20-25 cm) for July and September, and 5 depths (0-5 cm, 5-10 cm, 10-15 cm, 15-20, and 20-25 cm) for August). The remaining samples were collected in prior years and are described in main text in supplemental tables.

Library preparation and sequencing were performed at the Department of Energy Joint Genome Institute using standard methods. Plate-based RNA sample prep was performed on the PerkinElmer Sciclone NGS robotic liquid handling system using Illumina Ribo-Zero rRNA Removal Kit (Bacteria) and the TruSeq Stranded Total RNA HT sample prep kit following the protocol outlined by Illumina with the following conditions: total RNA starting material of 100 ng per sample and 10 cycles of PCR for library amplification. The prepared libraries were quantified using KAPA Biosystem's next-generation sequencing library qPCR kit and run on a Roche LightCycler 480 real-time PCR instrument. Sequencing of the flow cell was performed on the Illumina NovaSeq sequencer using NovaSeq XP v1 reagent kits, S4 flow cell, and following a 2x150 indexed run recipe. For the remaining 24 samples, these were previously reported in (1, 3), with 12 collected in 2014 and 12 from 2015.

Two samples did not yield sufficient biomass to be sequenced at JGI. For these two samples and nine additional samples from 2015, RNA-seq library construction and sequencing was performed at The Genomics and Microarray Shared Resource at University of Colorado Denver Cancer Center, Denver, CO, USA. Library preparation and rRNA depletion were performed using the Zymo-Seq Ribo Free Total RNA Library Kit Cat No. R3000 and libraries were sequenced on the Illumina NovaSeq 6000 using 2x150 paired end reads on an S4 flow cell.

Genome assembly and binning.
Metagenomic samples were assembled and binned using multiple approaches to maximize assembly and metagenome assembled genome (MAG) recovery **(11)**. Collectively, this resulted in 42 samples for the genome-resolved database made up of 2,502 unique MAGs, with assembly and binning methods described below. Additionally, the 2,502 dereplicated MAG database is available on Zenodo (see files MAGs.zip). Reported more extensively in **Supplementary Table 1**, but briefly here MAGs were recovered from

1) 2013 soil field samples (n=9), sequencing and selected MAGs published previously (2, 12)
2) 2014 soil field samples (n=3), sequencing and selected MAGs published previously (1, 3)
3) 2015 soil field samples (n=3), sequencing and selected MAGs published previously (1, 3)
4) 2018 soil field samples, *released in this study* (n=16)
5) lab enrichment samples, sequencing and selected MAGs published previously (n=2) (4)
6) lab enrichment samples, *released in this study* (n=9)

Raw metagenomic reads from the 2018 metagenomes were trimmed using Sickle (pe) (13) and then full assemblies were generated using Megahit (v1.2.2) (14) with parameters --k-min 41 --k-max 121 --k-step 10. Subsampled assemblies using 30% of sequencing reads were generated using IDBA-UD v 1.1.3 (15) with default parameters. To generate more finely resolved contig depth signatures as an aid to binning, reads from multiple samples were mapped to each assembly using BBMap with minid=0.95 and ambig=random (v 38.70) (16) and these profiles were used with assembled contigs for binning using

Metabat2 (17). Bins from these assemblies were combined with bins from metagenomic assemblies derived from earlier sampling of this wetland and from metagenomic sequencing of wetland soil enrichments (1, 3, 4).

This bin pool was dereplicated using dRep (v 1.4.3) (18) at 99% identity with the 'winning' bins retained as a preliminary wetland genome database. Finally, reads from the 2018 assemblies (n=16) that did not map to contigs in the preliminary MAG database were grouped by depth (e.g. D1-D3 and D4-D5) and co-assembled using Megahit (14). These two co-assemblies were binned as described above and dereplicated with the preliminary database yielding a final database of 3217 wetland MAGs. MAG completeness and contamination was estimated using CheckM (19) and taxonomy assigned using GTDB-tk (v2.4.0) with GDTB database r220 (20, 21). Of these 3217 MAGs, 2502 were of medium or high quality based on adapted MIMARKS standards (completeness >=50% and contamination <10%) (22).

Assessment of genome transcription. Raw metatranscriptomic reads were filtered and initial QC performed using Joint Genome Institute standard protocols. Filtered reads were further quality trimmed using sickle (pe) (13) and mapped to the full MAG database using BBMap (v 38.70) (16). Following mapping, bamfiles were filtered to retain mappings of at least 99% ID and per-gene read counts were estimated using HT-Seq (v0.11.2) (23). The read count table was first filtered to retain genes which have a minimum total count of 10 across all samples. The filtered table was normalized for gene length and TMM normalized using the EdgeR 'cpm' function with log2 normalization (24). MAG mean transcription values were calculated for those MAGs having at least 20 genes transcribed. Due to file size constraints, the per MAG transcript profiles across 133 metatranscriptome samples are accessible in the zenodo library that accompanies this manuscript (10.5281/zenodo.8194033). This includes the MAG based analysis (**owc_metat_table_mags.csv)** as well as annotated expressed DRAM genes **(owc_metat_table_mags_genes.csv).**

To calculate the percentage of a genome transcribed for the 1,948 genomes recovered where at least 20 genes were transcribed, we first counted the number of genes transcribed per genome, and then divided that count by the number of predicted genes per genome as estimated by CoverM (25) and multiplied by 100. We performed a Mann-Whitney U test to assess if there was a significant difference in the percentage of a genome expressed in bacterial versus archaeal genomes. We also classified phyla by their median transcription and variability by calculating the median percentage of genomes transcribed (summarized by phylum) and by the variability of that % transcription of MAGs within a phylum, calculated as the median relative standard deviation (RSD, aka coefficient of variation). These data are accessible via the zenodo.

MAG and Gene Based Functional profiling. Bacterial and archaeal MAGs were annotated using DRAM v1(26). Because of file size constraints, these data files are included on the zenodo (see data accessibility). This includes (1) the MAG amino acid gene sequences derived from DRAM gene calls (fasta file) as **OWC_HQMQ_DB_genes.faa.gz** and MAG DRAM annotations as **OWC_HQMQ_DB_ANNOTATIONS_20220208.txt.gz.** We also annotated bacterial and archaeal metagenome-resolved transcriptomes *via* DRAM (26), with these also on zenodo also, as (1) metatranscriptomic expression per gene across 133 metatranscriptomes (csv table) in file **owc_metat_table_mags_genes.csv** and (2) the corresponding DRAM annotations for these transcribed genes in file **owc_metat_table_mags_genes_annotations.csv**.

To assign traits to MAGs, we curated the MAG annotations made by DRAM (26) using a ruleset visualized in **Supplementary Fig. S2**. MAGs were assigned potential traits derived from metagenomics and expressed traits derived from metatranscriptomics. From metagenomics, MAGs were first classified by respiratory capacity based on the presence of >50% of the subunits required for Complex 1 of the electron transport chain and the presence of at least one gene for an electron acceptor. Based on which

acceptor was predicted, a MAG would be assigned to a particular functional guild (e.g., N reducer, aerobic, iron reducer). As an example, for a MAG to be classified as microaerophillic, we required the MAG to have more than 50% of Complex 1 subunits and at least one subunit of a high affinity cytochrome oxidase. Likewise, if a MAG did not have more than 50% of the subunits required for Complex 1 of the electron transport chain or the potential for any electron acceptor, it was classified as an obligate fermenter.

Beyond respiratory metabolisms, other traits were assigned based on functional gene presence. For example, N fixation was assigned based on the presence of at least one subunit of any nitrogenase (**Supplementary Table S6**). For specific metabolisms, such as methanogenesis and methanotrophy, assignment was based on the presence of a key functional gene (*mcrA* and *pmoA*, respectively) or by manual curation including taxonomic information given the conserved nature of these metabolisms at the genus level and the tendency for these key functional genes to be on short contigs, precluding them from binning (1, 3). From metatranscriptomics, trait calls were based on potential calls from MAGs, but also required various levels of transcription to be considered expressed. For example, to be considered an expressed microaerophile, a MAG had to 1) be classified as a microaerophile based on metabolic potential (more than 50% of complex 1 subunit and at least one subunit of a low affinity cytochrome oxidase) and 2) had to be expressing more than 30% of complex 1 subunits and at least one subunit of a low affinity cytochrome oxidase. For trait classified based on an absence of data (obligate fermenter) or taxonomy (methanogen, methanotroph), MAGs needed to be considered active (more than 20 genes transcribed) to be classified as expressed for a particular trait. As trait assignment was based on MAG annotation, we also assigned MAG-level transcription. Likewise, MAGs could be assigned to more than one functional guild, and totals were summed across samples. The metabolic guild assignments for the 2,502 MAGs in both potential and expression is contained in **Supplementary Table S6**.

To minimize potential bias associated with incomplete or contaminated MAGs, all genomes were manually curated prior to functional assignment, and gene calls were restricted to high-confidence contigs. Functional guild assignments were based on the presence of expressed marker genes rather than absence alone, and taxonomy was used as an independent consistency check to confirm biological plausibility. Importantly, downstream trait-based analyses relied on metatranscriptome mapping, ensuring that functional interpretations were grounded in observed gene expression.

Additionally, we note that functional gene based phylogenetic analyses were performed to refine the annotation of nitrogen related metabolism including genes annotated as respiratory nitrate reductase (*nar*), nitrite oxidoreductase (*nxr*), ammonia monooxygenase (*amo*), or methane monooxygenase (*pmo*) to improve the assignment the nitrogen cycling capabilities of MAGs (**Supplementary Table S6**). Specifically, Nxr/Nar and PmoA/AmoA amino acid reference sequences were downloaded and this set of reference sequences were combined with amino acid sequences of homologs from the MAGs recovered here, aligned separately using MUSCLE (v3.8.31) (27), and run through an in-house script for generating phylogenetic trees. This resulted in two phylogenies, one for *Nxr/Nar* genes and one for *Pmo/Amo* genes, with this approach used to refine the homology-based gene annotations in the MAG database.

For MAGs assigned to the methanogen functional guild, substrate classification was further characterized, assigning each as hydrogenotrophic methanogen, acetoclastic methanogen, or methylotrophic (methyl-N, methyl-S, methyl-O) methanogen, with genes required for each detailed in **Supplementary Table S11**. Gene calls were based on DRAM annotations. Specifically, all genes involved in methanogenesis were inventoried per MAG and then based on those genes that were specific for a substrate type, substrate calls were made. Archaeal metabolism calls were based on DRAM annotations with specific genes required for each detailed in **Supplementary Table S8** (Archaea specific).

Identification of major environmental axes driving wetlands microbiome variation. We first reduced collinearity by dropping a subset of geochemical and climatic variables that were highly correlated with retained variables (Spearman's r > 0.75; **Supplementary Fig. S3**) including Ethanol, Isopropanol, extractable Na, extractable Al, % Sand, extractable K, and temperature. We log-transformed variables where appropriate and standardized variables prior to analyses. Our final set of environmental predictors included measured soil properties (Dissolved Oxygen, Acetate, Nitrate, Nitrite, Sulfate, Fe2, pH, CEC, % Total Organic Carbon, % Silt, % Clay, Hydraulic Conductivity, extractable Mg), site attributes (ecotype, depth, collection month, collection year), associated geographic variables (latitude/longitude), and greenhouse gas concentrations (CH_4 and CO_2 peeper concentrations).

To characterize how the composition and transcription of microbial communities differed across time, space, and major geochemical gradients, we first assessed calculated dissimilarities with Bray-Curtis metric on square root transformed relative abundances. We used three metrics of microbiome communities: taxonomic composition (ASVs), relative mean MAG transcription, and relative mean metabolic guild transcription, and visualized as ordinations with nonmetric multidimensional scaling (k=3). We tested the correlation between pairwise distances in bacterial and archaeal composition/transcription to pairwise distances in our suite of geochemical and spatiotemporal variables using Mantel tests with 999 permutations. Then, to identify the best overall explanatory models associated with differences in composition and expression we conducted multiple regression analyses on distance matrices (**Supplementary Fig. S3**).

Genome co-expression network analysis and process-based model generation. Beyond individual lineages correlations described above, we were interested in identifying if there were community-level emergent properties (e.g., clusters of co-transcribed genomes) that were highly correlated in their relative expression to relate to in situ measurements of greenhouse gas concentrations (CH_4, CO_2). To test this, we used weighted correlation network analysis (WGCNA) (28) across all of our metatranscriptomes (n=132, 1 sample was excluded due to outlier screening within the WGCNA pipeline). A signed adjacency measure for each pair of features was calculated with the power threshold of 7, identified by plotting mean connectivity as a function of the soft-thresholding power and selecting the power threshold above 0.80 to optimize the scale-free topology network fit. The adjacency matrix was used to calculate topological overall measure (TOM) and derive a co-expression network. We used a signed hybrid network, with a minimum module size of 50, and a merge height of 0.3. Hierarchical clustering was employed to resolve subnetworks (i.e., modules), using a distance based on the calculated TOM. For each subnetwork, we calculate pairwise Pearson correlation coefficients between the subnetwork principal components and key geochemical measurements (pH, sulfate, CEC, nitrate, nitrite, DO, % TOC, conductivity, depth, Fe_2, acetate, Mg, and ecotypes) as well as our *in situ* measurements of CO_2 and CH_4 concentrations.

We next assessed the two subnetworks that had the highest significant correlation scores (correlations between module eigenvalues and CO_2/CH_4 concentrations, which we refer to as 'traits') with an intramodular analysis. Our first goal was to identify genomes with a high genome trait significance (the absolute value of the correlation between the genome and the trait) and subnetwork membership (the correlation of the module eigenvalue and the genome expression profile). Second, we also computed VIP scores from sPLS (29) models (in relation to both CH_4 and CO_2) for all genomes within each of the two subnetworks to obtain an additional metric of their individual importance. VIP scores were highly correlated with genome trait significance scores (rho > 0.9 for all). Subnetwork #1 contained 372 genomes (the subnetwork–trait correlation to CH_4 and CO_2 was 0.38 and 0.36 respectively, with $P<0.0001$ for both). Likewise, for each subnetwork, we found that genome significance to be significantly correlated to subnetwork membership both for CH_4 (Rho = 0.54 and 0.52 for subnetworks #1 and 2, respectively; $P<0.001$) and for CO_2 (Rho = 0.54 and 0.29 for subnetworks #1 and 2, respectively; $P<0.001$).

MAGs within the process-based model were based on WGCNA (28) and sPLS (29) analyses, as well as MAG expression profiles. First, MAGs within each of the two subnetworks (brown and turquoise) were filtered to those with a VIP score ≥ 1 in relation to CH_4 and then clustered at the genus level. Then clusters were classified based on expressed traits. For clusters containing more than one MAG, functional guilds were consolidated. If trait assignments disagreed (e.g. aerobe and obligate fermenter) within a cluster, the functional guild from the most complete MAG within a cluster was used. Clusters with no expressed adjectives or without at least one MAG expressing ≥ 20% of its genes were removed from subsequent analyses. Remaining clusters were retained in the process-based model. For MAGs within the remaining clusters, DRAM annotations were used to profile expressed carbon usage. Based on the expressed carbon utilization and functional guild of each cluster, a schematic representation of the process model (**Fig. 6E**) was constructed in Adobe Illustrator. All the statistics and metabolic calls used to construct Fig. 8 are provided in **Supplementary Tables S12 and S13**, including VIP values, expression patterns, functional guilds, and carbon utilization provided in. This analysis workflow is shown in **Fig. S7**.

Figure S1. Experimental Design. (A) Upset plot showing the major data types obtained from samples and the number of samples obtained for each set (top bars) including from left to right: geochemistry, 16S rRNA amplicon sequencing, NMR metabolomics, metatranscriptomics, and metagenomics. The gray bars (right) represent the number of interesting sets across sample types. **(B)** Overview of the number of metagenome-resolved genomes (MAGs) recovered in this study. All 2,502 medium and high-quality (MQHQ) MAGs used in analyses were ≥50% complete, were <10% contaminated. **(C)** Boxplot highlights the amount of sequencing in Giga basepairs (Gbp) for this study in Old Woman Creek (OWC) versus other classified wetlands in the Sequence Read Archive (SRA) for metagenomes and metatranscriptomes. Each point represents a single sample, and boxplots show the median Gbp. **(D)** Overview of the genome-resolved metagenomics (top) and genome-resolved metatranscriptomics (bottom) bioinformatics pipeline we developed to obtain genome-resolved microbial community activity profiles. The experimental data described in this figure can be accessed in **Supplementary Table S1, S4**. **(E)** Sampling design summarizes site-level sampling across multiple years (2013, 2014–2015, and 2018) at Old Woman Creek. Top panels show aerial maps of sampling locations across wetland zones (mud, plant, and open-water habitats) and conceptual diagrams of sediment core collection and water–air interface measurements. Lower panels detail the temporal sampling structure by campaign (October 2013; November 2014, February 2015, May 2015, August 2015; and May–September 2018). Colored circles indicate analyses performed on each core and depth, Replication structure, habitat type, and analytical coverage are indicated for each transect and time point.

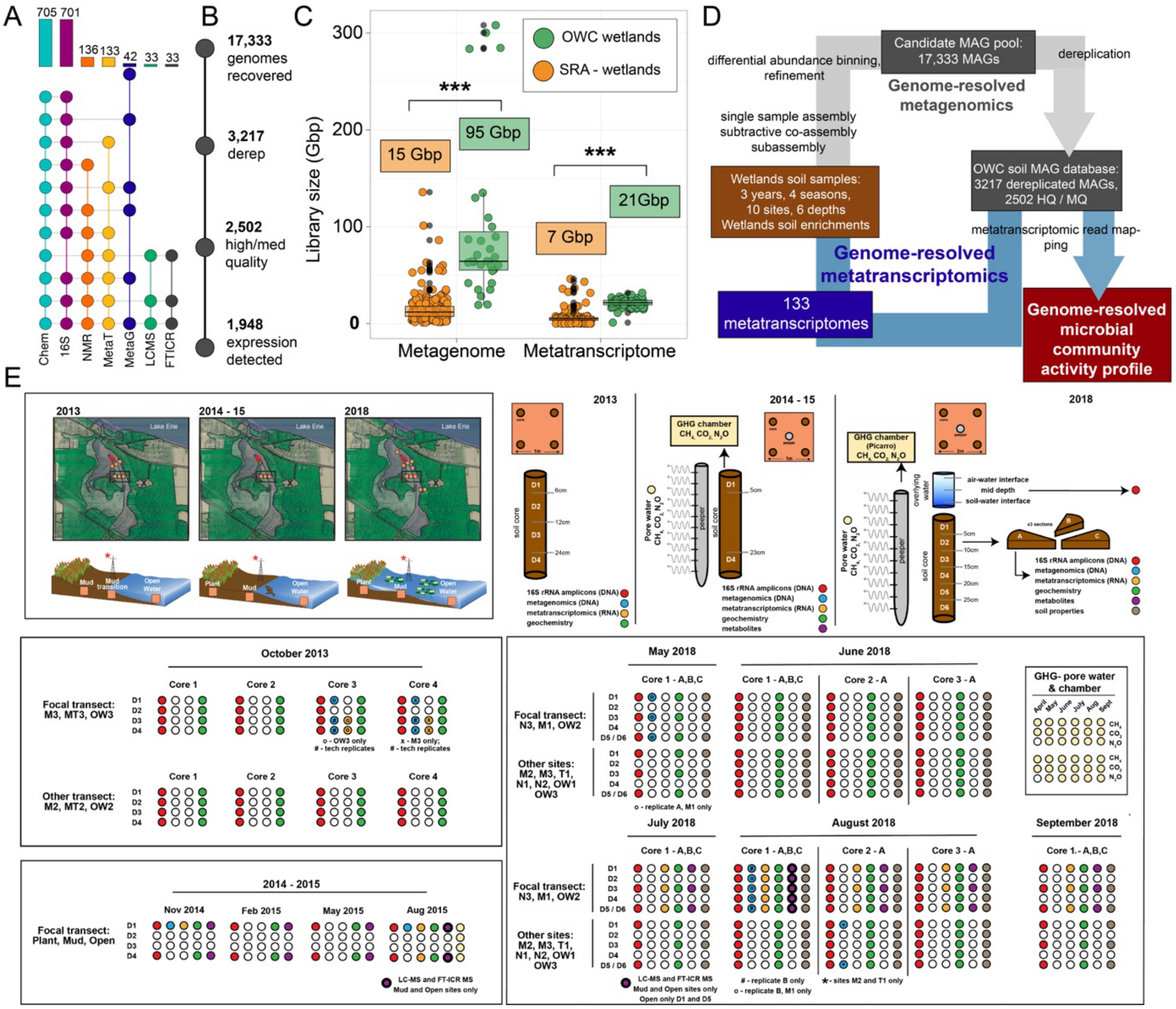

Figure S2. Overview of workflow for assigning MAGs to biogeochemical processes. The 2,502 metagenome assembled genomes were characterized for functional potential and expression of 14 major biogeochemical contributions. Here we show how we used gene content to classify the aerobic, microaerophilic, photosynthetic, Nitrogen (N) reducer, Iron (I) reducer, Sulfur (S) reducer, methanogen, obligate fermenter, Dissimilatory Nitrite Reduction to Ammonium (DNRA), nitrogen (N) fixation, nitrification, aerobic methanotroph, Iron oxidizer, and Sulfur (S) oxidizer. The path diagram summarizes the gene suites required for a biogeochemical classification. For the subset of 1,948 transcribed genomes, we also assigned 'expressed' metabolism from transcripts recruited back to MAGs. For some genes (e.g., *pmoA*), phylogenetic analyses and other non-homology-based methods were used for confirmation. Additional details are provided in the methods section, **MAG and Gene Based Functional profiling,** while the data derived from this approach is included in **Supplementary Table S6.**

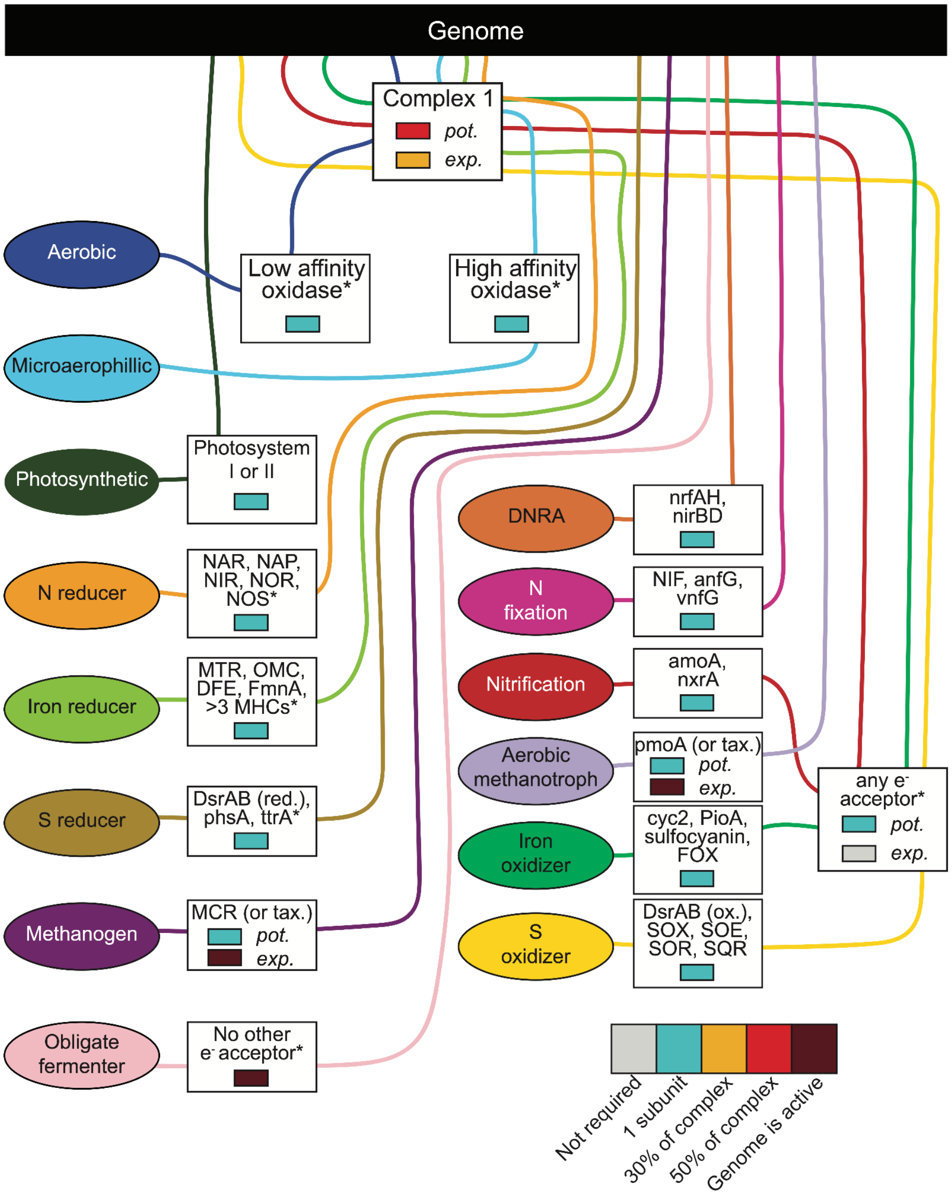

Figure S3. Spatiotemporal predictors of microbial community dynamics. (A) Correlation analysis highlights the soil, porewater, and flux metadata associated with metatranscriptomics and metagenomics samples, and the correlation of these variables to one another (denoted in heatmap legend). The data supporting this figure is included in **Supplementary Table S4** and includes the year, month, land coverage, depth, core, site transect. **(B)** PERMANOVA derived bar plots visualize the relative importance of spatiotemporal predictors included in the models for explaining the overall variation of composition, transcription, and guild microbiome information. **(C)** Mantel based bar plots visualize the mantel correlations of geochemical predictors included in multiple regression models for explaining overall variation. The model output data from PERMANOVA and mantel tests reported here is included in **Supplementary Table S7**.

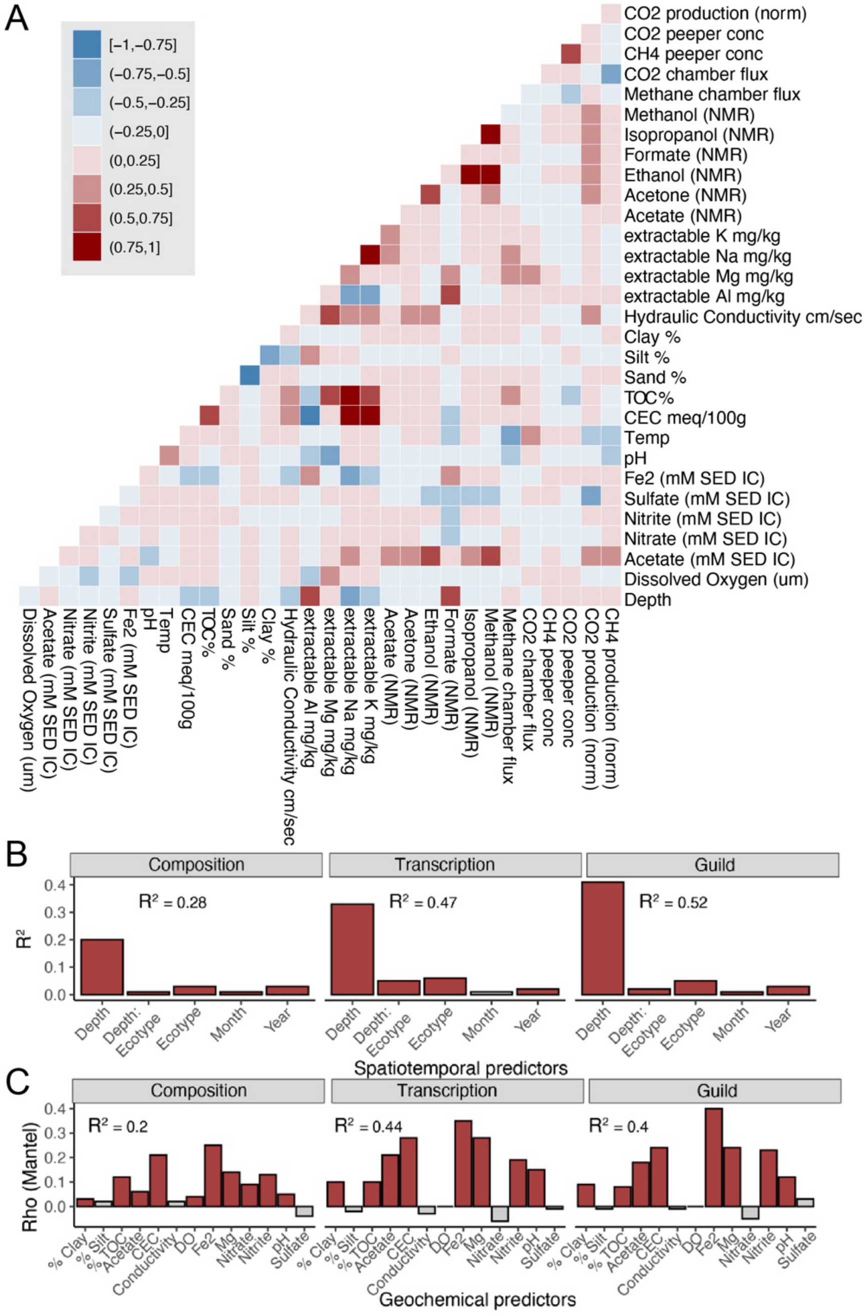

Figure S4. Methanogen MAG taxonomy, function, and expression. Alluvial plot highlighting the taxonomic diversity of methanogen MAGs recovered here. The first gray bar chart summarizes the number of genomes per genus recovered and the second gray bar chart summarizes the max transcription detected of a MAG within the genus. The MAG-resolved metabolisms show transcription-based substrate utilization.

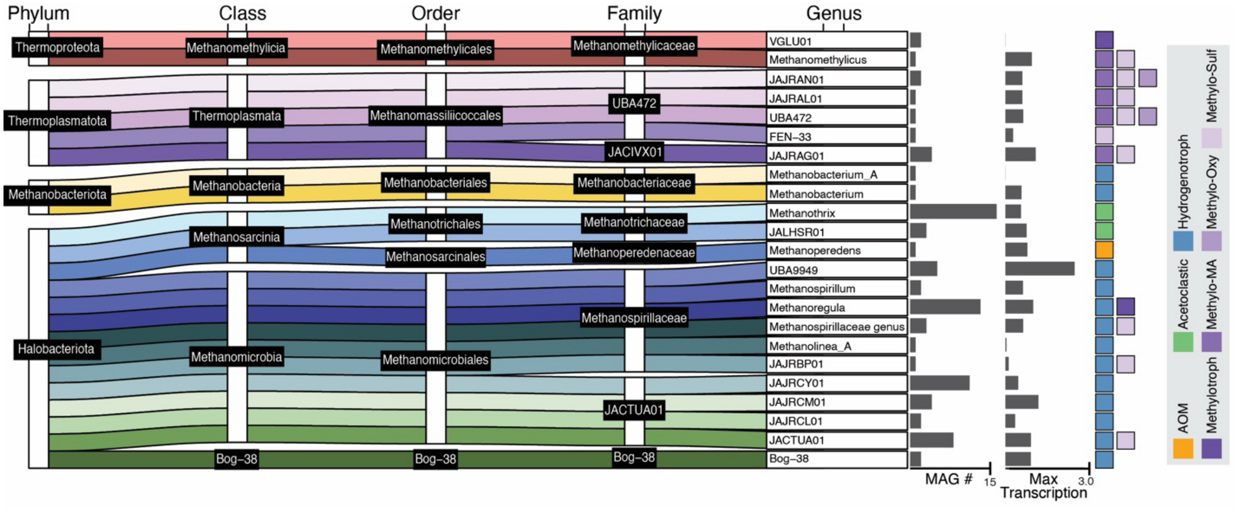

Figure S5. Methanogen substrate concentrations across depths. (A) Top panels show porewater concentrations (µM) of methanogen substrates (formate, acetate, and methanol) measured by NMR across five depth intervals (D1: 0–5 cm; D2: 5–10 cm; D3: 10–15 cm; D4: 15–20 cm; D5: 20–25 cm). Bottom panels show LC–MS derived abundances (log_2 peak area) potential methylotrophic methanogenic substrates. Boxplots represent the distribution across replicate cores at each depth, with center lines indicating medians and whiskers showing variability. Chemical structures are shown, with carbons utilized for methane generation highlighted by red circles. **(B)** Boxplots show log transformed NMR concentrations of acetate, formate, and methanol by year.

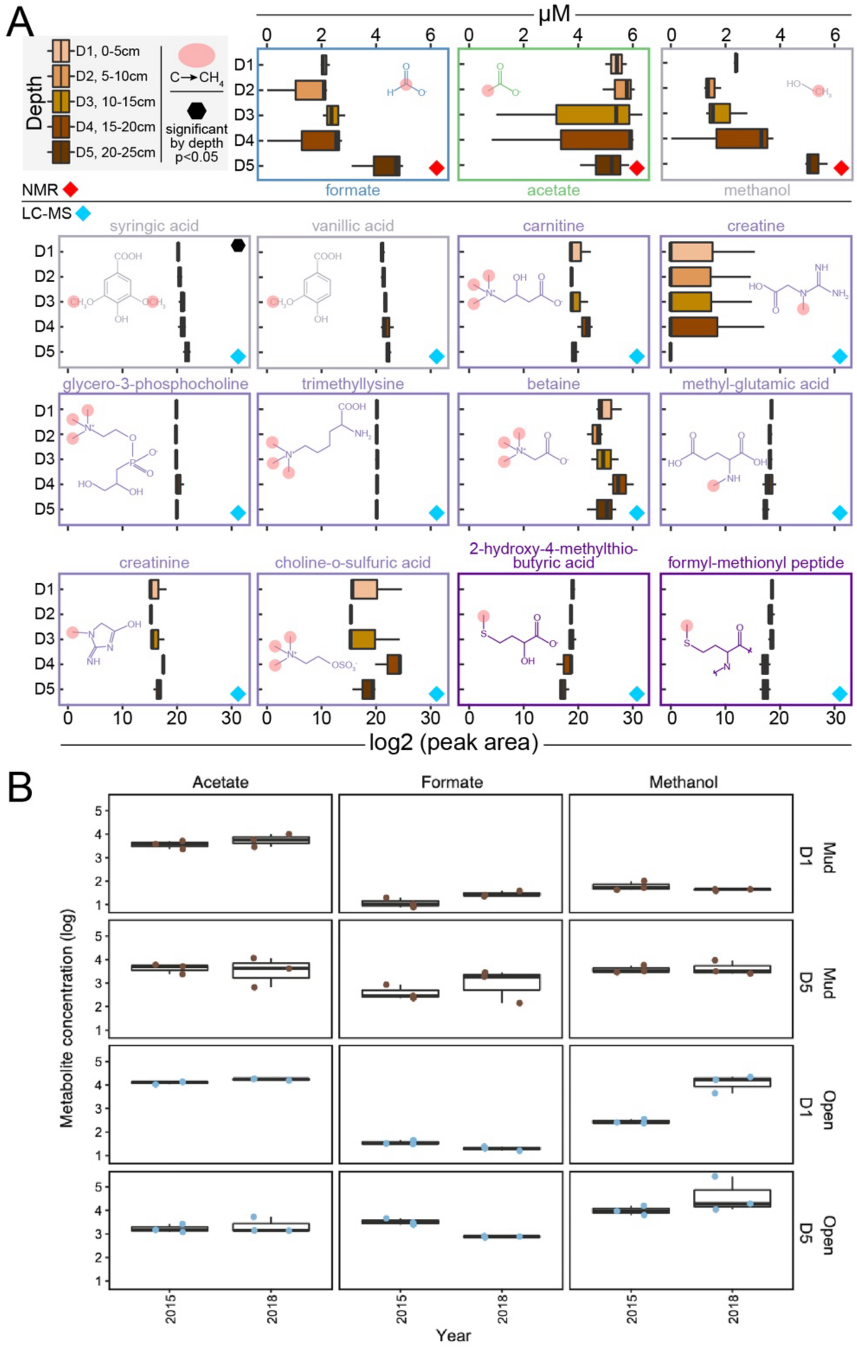

Figure S6. Methane cycling microorganisms show coordinated gene expression patterns, with groups co-localized by depth neighborhoods. By row metatranscriptome data from 3 cores at 3 depths (n=9) for each month (July, August, September) in blocks. Each column is the mean genome transcript for methanotroph or methanogen MAGs, with taxonomy and naming indicated on bottom and energy strategy denoted by box colors. Transcript response patterns are clustered by location, with some methanogens active in all depths in all seasons, some showing stronger surface, surface-middle, deep, deep-middle, and no clear depth signal. The data used to construct this figure is located in Supplementary Table S10 and on the zenodo DOI under file **owc_metat_table_mags.csv**.

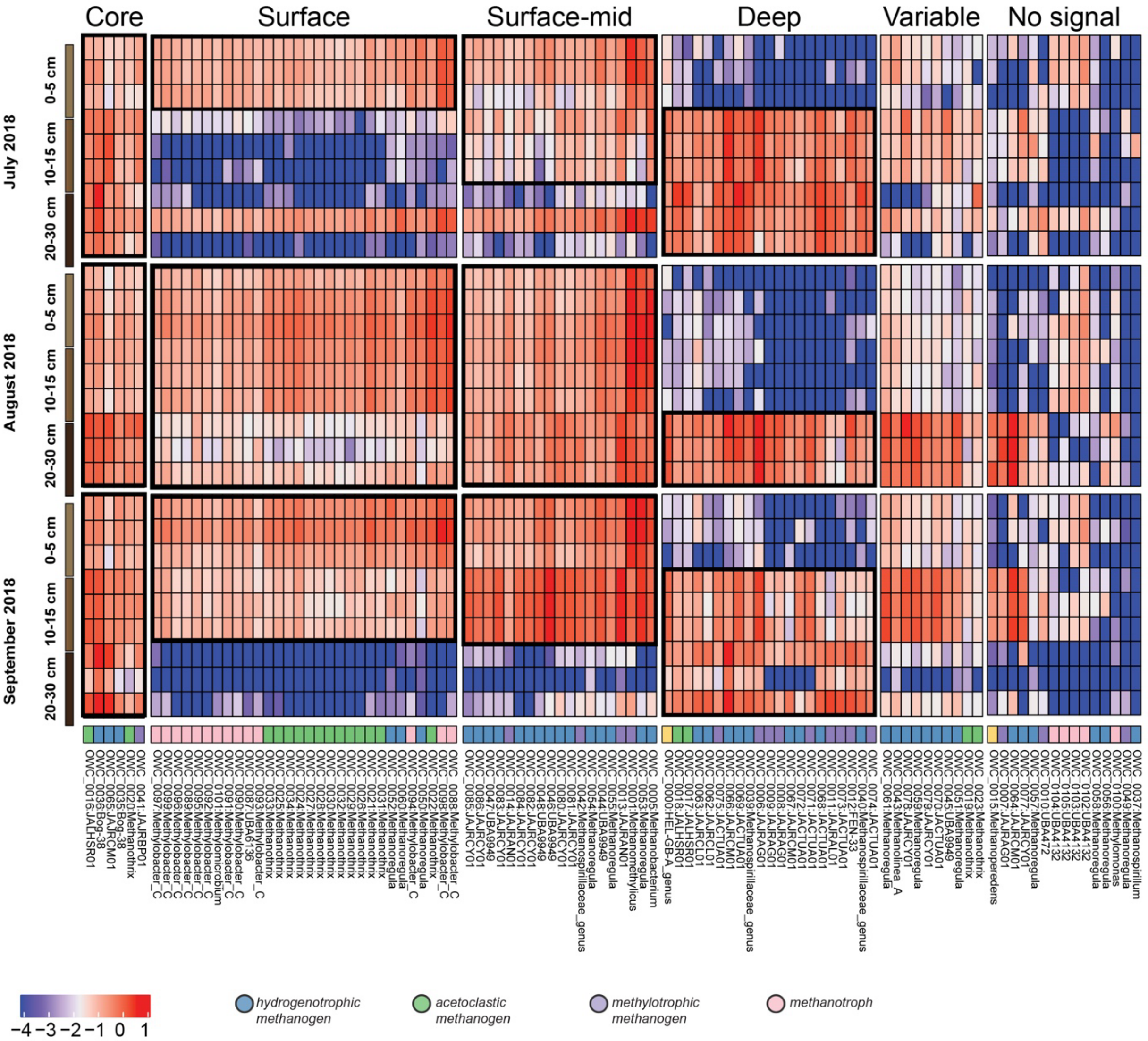

Figure S7. Workflow for data integration to identify functional guilds that predict soil methane. The left most column highlights the biological questions addressed (Questions). The middle column shows the actual data from other parts of this manuscript used to answer the question (Data/Input). The third column/arrow shows the statistical method used (Method). The fourth, most right column, shows the outcome of each analysis (Output). These output figures correspond to main text Figure 6. We denote the main text and supplementary figures referenced in the data inputs and outputs columns. Data supporting this figure is included in **Supplementary Tables S12** and **S13**.

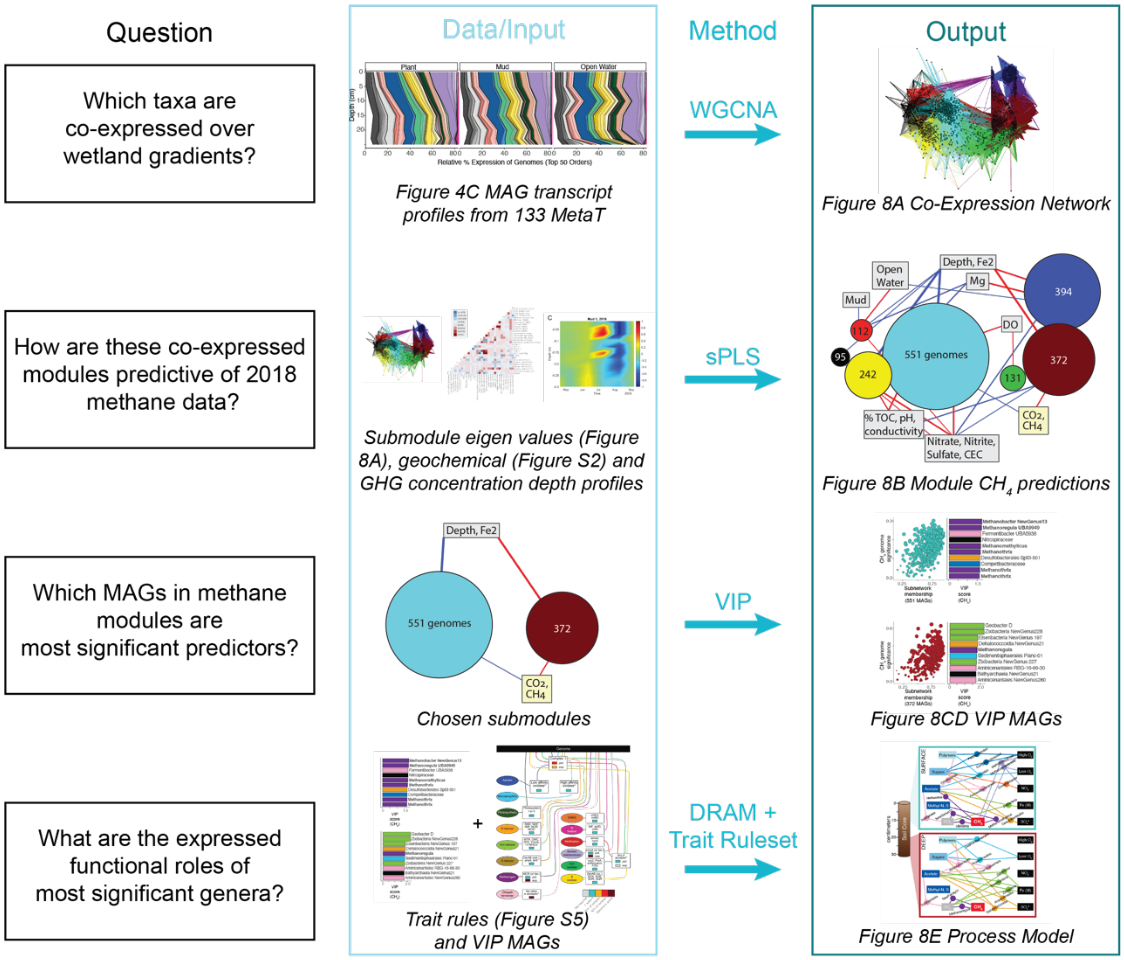

Captions for Supplementary Tables S1-S13. The bolded pre-fix relates to the spreadsheet worksheet name.

Table S1. OWC_Samples: Overview and source details of the Old Woman Creek samples included in this study, including metagenomes, metatranscriptomes, and 16S rRNA gene amplicon sequences. For all metagenomes and metatranscriptomes, the corresponding NCBI accession number and sequencing depth (in Gbp) is provided.

Table S2. 16S: The 16S rRNA amplicon sequencing data from 671 samples. This includes the amplicon sequencing variants (ASV) and relative abundances across space and time. Bacterial and Archaeal taxonomic assignments are listed for each ASV.

Table S3. The bin stats: Data for the 2,502 medium and high-quality MAGs recovered and their attributes, including: the final database ID, a reference bin ID (includes sample bin was recovered from), the RED taxonomy from GTDB (including assigned novelty status), GTDB-Tk assigned taxonomy, contamination, completeness, genome size, number of scaffolds, number of contigs, N50 scaffolds, N50 contigs, % GC, coding density, predicted genes.

Table S4. MetaT metadata: Overview of the metadata associated with metatranscriptomics and metagenomics samples, including year collected, month collected, ecotype code, depth, core, site transect, associated geochemistry (pH, DO, CEC etc.), NMR determined metabolites, as well as peeper concentration and flux measurements.

Table S5. Most transcribed and ubiquitous taxa: Data for panel **Fig. 1C**, including Max Transcription, Mean Transcription, and number of samples where transcribed.

Table S6. Bin_adjectives: The metabolic guild assignments for 2,502 MAGs in terms of both functional potential (e.g. metagenomic annotations) and transcribed potential (e.g. metatranscriptomic annotations, available for the subset of 1,948 MAGs that met our minimum transcription threshold), corresponding with **Fig. 2**.

Table S7. Environmental_drivers: Model outputs (mantel tests and PERMANOVA models) assessing the relationships between wetlands microbial communities (including the composition, transcription, and guild level) and spatiotemporal and geochemical variables to explain variation in the distribution of relative abundance. The R^2 values reported refer to PERMANOVA predictors and rho values refer to mantel tests (see Methods for details), with the data reported in **Fig. 3** and **Supplementary Fig. S3**.

Table S8. Archaeal_Annotation_Calls: Functional assignments for Archaeal genomes, and gene-level requirements for assignments corresponding with **Fig. 4**.

Table S9. Metabolites: Soil metabolite concentrations as detected by LC-MS and NMR that are known and possible methanogen substrates. These data were collected across 2015 and 2018 from surface and deep cores. These data are reported in **Supplementary Fig. S5**.

Table S10. LCMS Metabolite annotation: Metabolite annotation data including specifics of LC-MS methodology. This information was used to generate the information in Table S8.

Table S11. Methanotroph_and_methanogen annotations: Genome inferred substrate use for methanogens and methanotrophs as defined by gene content. This table includes the gene-based rule set that profiled 80 genes to assign the substrate use categories from metagenome and metatranscriptome data in this study. These data are reported in **Fig. 4**.

Table S12. WGCNA_traits: Correlations between transcription of bacterial and archaeal genomes and geochemical attributes (pH, depth, total organic carbon, ecotype, etc.) detected via WGCNA network construction and analysis for each module detected.

Table S13. Process_model_map: Genomes included in focal WGCNA subnetworks (brown and turquoise) used to build process models (**Fig. 6E**). For each circle labeled in **Fig. 6E**, this table includes specific genomes in each transcriptional module, full taxonomy string, figure taxonomy label (column Label Name for **Fig. 6**), corresponding VIP scores and metabolism (redox guild, consumption, production, C fixation, sugars utilization, polymers, hemi-cellulose, dimers, short chain fatty acids, pyruvate to acetyl-coA).

In addition, we direct readers to the Zenodo DOI 10.5281/zenodo.8194033 (see **Data Availability**). This zenodo data set contains the following files, which are too large or not the appropriate format (excel format) to be included as supplementary table.

1. **MAGs.zip** Dereplicated database of 2,502 MAGs (fasta files)
2. **owc_HQMQ_DB_genes.faa.gz** MAG amino acid gene sequences derived from DRAM gene calls (fasta file)
3. **owc_HQMQ_DB_ANNOTATIONS_20220208.txt.gz** MAG DRAM annotations
4. **owc_metat_table_mags.csv** Metatranscriptomic expression per MAG across 133 metatranscriptomes (csv table)
5. **owc_metat_table_mags_genes.csv** Metatranscriptomic expression per gene across 133 metatranscriptomes (csv table)
6. **owc_metat_table_mags_genes_annotations.csv** corresponding DRAM annotations to #5 for transcribed genes (csv table)

SI References

1. J. C. Angle, *et al.*, Methanogenesis in oxygenated soils is a substantial fraction of wetland methane emissions. *Nat Commun* **8**, 1567 (2017).

2. A. B. Narrowe, *et al.*, High-resolution sequencing reveals unexplored archaeal diversity in freshwater wetland soils. *Environ Microbiol* **19**, 2192–2209 (2017).

3. G. J. Smith, *et al.*, Members of the Genus Methylobacter Are Inferred To Account for the Majority of Aerobic Methane Oxidation in Oxic Soils from a Freshwater Wetland. *mBio* **9**, e00815-18.

4. A. B. Narrowe, *et al.*, Uncovering the Diversity and Activity of Methylotrophic Methanogens in Freshwater Wetland Soils. *mSystems* **4**, e00320-19 (2019).

5. J. B. Ellenbogen, *et al.*, Methylotrophy in the Mire: direct and indirect routes for methane production in thawing permafrost. *mSystems* **0**, e00698-23 (2023).

6. J. G. Caporaso, *et al.*, Ultra-high-throughput microbial community analysis on the Illumina HiSeq and MiSeq platforms. *ISME J* **6**, 1621–1624 (2012).

7. E. Bolyen, *et al.*, Reproducible, interactive, scalable and extensible microbiome data science using QIIME 2. *Nat Biotechnol* **37**, 852–857 (2019).

8. B. J. Callahan, *et al.*, DADA2: High-resolution sample inference from Illumina amplicon data. *Nat Methods* **13**, 581–583 (2016).

9. C. Quast, *et al.*, The SILVA ribosomal RNA gene database project: improved data processing and web-based tools. *Nucleic Acids Res* **41**, D590-596 (2013).

10. M. Borton, *et al.*, Targeted curation of the gut microbial gene content modulating human cardiovascular disease. *mBio* **14**, e0151123 (2023).

11. M. A. Borton, *et al.*, A functional microbiome catalogue crowdsourced from North American rivers. *Nature* **637**, 103–112 (2025).

12. A. B. Narrowe, *et al.*, Complex Evolutionary History of Translation Elongation Factor 2 and Diphthamide Biosynthesis in Archaea and Parabasalids. *Genome Biology and Evolution* **10**, 2380–2393 (2018).

13. najoshi, sickle - A windowed adaptive trimming tool for FASTQ files using quality. (2023). Deposited 16 June 2023.

14. D. Li, C.-M. Liu, R. Luo, K. Sadakane, T.-W. Lam, MEGAHIT: an ultra-fast single-node solution for large and complex metagenomics assembly via succinct de Bruijn graph. *Bioinformatics* **31**, 1674–1676 (2015).

15. Y. Peng, H. C. M. Leung, S. M. Yiu, F. Y. L. Chin, IDBA-UD: a de novo assembler for single-cell and metagenomic sequencing data with highly uneven depth. *Bioinformatics* **28**, 1420–1428 (2012).

16. B. Bushnell, “BBMap: A Fast, Accurate, Splice-Aware Aligner” (Lawrence Berkeley National Lab. (LBNL), Berkeley, CA (United States), 2014).

17. D. D. Kang, *et al.*, MetaBAT 2: an adaptive binning algorithm for robust and efficient genome reconstruction from metagenome assemblies. *PeerJ* **7**, e7359 (2019).

18. M. R. Olm, C. T. Brown, B. Brooks, J. F. Banfield, dRep: a tool for fast and accurate genomic comparisons that enables improved genome recovery from metagenomes through de-replication. *ISME J* **11**, 2864–2868 (2017).

19. D. H. Parks, M. Imelfort, C. T. Skennerton, P. Hugenholtz, G. W. Tyson, CheckM: assessing the quality of microbial genomes recovered from isolates, single cells, and metagenomes. *Genome Res.* **25**, 1043–1055 (2015).

20. P.-A. Chaumeil, A. J. Mussig, P. Hugenholtz, D. H. Parks, GTDB-Tk: a toolkit to classify genomes with the Genome Taxonomy Database. *Bioinformatics* **36**, 1925–1927 (2020).

21. D. H. Parks, *et al.*, Recovery of nearly 8,000 metagenome-assembled genomes substantially expands the tree of life. *Nat Microbiol* **2**, 1533–1542 (2017).

22. R. M. Bowers, *et al.*, Minimum information about a single amplified genome (MISAG) and a metagenome-assembled genome (MIMAG) of bacteria and archaea. *Nat Biotechnol* **35**, 725–731 (2017).

23. S. Anders, P. T. Pyl, W. Huber, HTSeq--a Python framework to work with high-throughput sequencing data. *Bioinformatics* **31**, 166–169 (2015).

24. M. Smid, *et al.*, Gene length corrected trimmed mean of M-values (GeTMM) processing of RNA-seq data performs similarly in intersample analyses while improving intrasample comparisons. *BMC Bioinformatics* **19**, 236 (2018).

25. B. J. Woodcroft, CoverM. (2023). Deposited 26 June 2023.

26. M. Shaffer, *et al.*, DRAM for distilling microbial metabolism to automate the curation of microbiome function. *Nucleic Acids Research* **48**, 8883–8900 (2020).

27. R. C. Edgar, MUSCLE: multiple sequence alignment with high accuracy and high throughput. *Nucleic Acids Res* **32**, 1792–1797 (2004).

28. P. Langfelder, S. Horvath, WGCNA: an R package for weighted correlation network analysis. *BMC Bioinformatics* **9**, 559 (2008).

29. H. Abdi, Partial least squares regression and projection on latent structure regression (PLS Regression). *WIREs Computational Statistics* **2**, 97–106 (2010).
